# Appraising the Genetic Makeup of an Allochthonous Southern Pike Population: An Opportunity to Predict the Evolution of Introgressive Hybridization in Isolated Populations?

**DOI:** 10.3390/ani13030380

**Published:** 2023-01-22

**Authors:** Marco Casu, Ilenia Azzena, Fabio Scarpa, Chiara Locci, Alessio Niffoi, Giovanni Battista Delmastro, Paolo Lo Conte, Antonio Varcasia, Stefano Bovero, Alessandro Candiotto, Daria Sanna, Piero Cossu

**Affiliations:** 1 Department of Veterinary Medicine, University of Sassari, 07100 Sassari, Italy; 2 Department of Biomedical Sciences, University of Sassari, 07100 Sassari, Italy; 3 Laboratorio di Ittiologia, Museo Civico di Storia Naturale, 10022 Carmagnola, Italy; 4 Funzione Specializzata Tutela Fauna e Flora, Città Metropolitana di Torino, 10122 Torino, Italy; 5 Independent Researcher “Zirichiltaggi” Sardinia Wildlife Conservation NGO, 07100 Sassari, Italy; 6 NaturaStaff Hydrobiologist, 14040 Mongardino, Italy

**Keywords:** biological invasions, introduced populations, southern pike, hybridisation, introgression, microsatellite markers

## Abstract

**Simple Summary:**

The southern pike is a freshwater top-predator fish that is endemic to rivers and lakes across Northern and Central Italy and is threatened by population decline and hybridisation with the northern pike. In this study, we focused on a southern pike population that has been recently introduced in an artificial lake, outside the species’ native range, and used genetic data to achieve a two-fold goal: (1) to investigate genetic diversity and (2) assess whether this population entirely consists of pure southern pike or is affected by hybridisation with the northern pike. The following conclusions were drawn from the results: (1) the genetic diversity of the introduced population is as large as that observed in wild populations, and (2) the presence of hybrid individuals is likely, which might be the descendants of old crosses between southern and northern pike. Several introduction events, a large number of founders, and hybridisation itself may explain the high genetic diversity that has been found in the introduced population. The present study may help further understand the genetic drivers of the successful establishment of introduced populations in new habitats. Moreover, the southern pike population from the Alto Flumendosa Lake may also be used as a test case to study how hybridisation evolves in isolated populations.

**Abstract:**

Biological invasions are a major threat to the conservation of biodiversity, as invasive species affect native biota through competition, predation, pathogen introduction, habitat alteration, and hybridisation. The present study focuses on a southern pike population, *Esox cisalpinus* (Teleostei: Esocidae), that has been introduced outside the species’ native range. Using microsatellite markers, this study’s objective was to gather baseline genetic information and assess the presence of hybrids between this species and *E. lucius* in the introduced population. The resulting estimates of genetic diversity and effective population size are comparable to those observed in the species’ native range. Although different methods yield contrasting and uncertain evidence regarding introgressive hybridization, the presence of late-generation hybrids cannot be completely ruled out. Large numbers of breeders as well as multiple introductions of genetically divergent cohorts and introgressive hybridisation may explain the high genetic diversity of this recently introduced southern pike population. The present study issues a warning that the conservation of southern pike’ introgressive hybridisation between northern and southern pike might be underestimated. The genetic information gathered herein may unravel the origin, number of introduction events, and evolutionary trajectory of the introduced population. This information may help us understand the evolution of introgressive hybridisation in the southern pike’s native areas.

## 1. Introduction

Biological invasions are now considered to be one of the major threats to biodiversity conservation as they may have wide ecological effects, such as affecting entire community structures [1,2]. Invasive species may impact native biota and increase the risk of the extinction of local populations through competition, predation, pathogen introduction, habitat alteration, and hybridisation [3,4,5,6]. Such negative effects may be enhanced in freshwater fish species that inhabit fragmented habitats and are characterised by small population sizes. In freshwater habitats, the introduction of non-native fish is often not accidental but fostered by recreative or economic interests, and hybridisation events between native and non-native fish are not rare [7,8]. For instance, for decades, the extensive stocking of depauperated populations with hatchery-reared fish has been a common practice used to counterbalance the decline of many wild fish populations due to overfishing and habitat deterioration (e.g., Splendiani et al. [8] and Larsen et al. [9]). However, stocking with hatchery-reared fry, which are often the offspring of non-local breeders, may jeopardise the genetic integrity of natural populations [10]. Hybridisation and genetic introgression, for instance, are among the most important factors affecting the native biodiversity of many Mediterranean freshwater fish communities [11,12,13].

This is also the case of the southern pike, *Esox cisalpinus* Bianco and Delmastro 2011, (syn. *E. flaviae* Lucentini et al. 2011) an esocid endemic to Italian freshwater habitats, which has recently been raised to the species rank as it is genetically and morphologically distinct from the northern pike, *E. lucius* [14,15]. As many other freshwater top predators, the southern pike is characterised by small population sizes and limited gene flow across drainage basins [16,17,18]. Genetic studies based on microsatellite loci evidenced moderate to strong genetic divergence among Italian populations, which likely depended on the smaller extent and higher fragmentation of Italian drainage basins compared to other European ones [16]. As the southern pike’s European counterpart, *E. lucius* [9,19], even *E. cisalpinus* populations are declining due to fishing pressure and habitat deterioration,, with decreases that reached peaks up to 90% in many localities [16,17]. Therefore, to halt this trend, restocking programs were enforced, which were based on the dissemination of hatchery-reared juveniles that were the offspring of wild-caught breeders [15,18]. The use of domesticated or wild hatchery stocks is commonly adopted to counterbalance fish population declines, but such programs rarely use genetic data to inform management practices [9,19]. Esocids are not an exception to this trend and one hundred years of transplantations without any information concerning the origin of individuals might have shadowed historical patterns of genetic variation and affected the contemporary ones [20]. For instance, extensive hybridisation between distinct genetic lineages was reported in many northern pike populations from Central Europe [21]. The picture is even worse for southern pike populations, as restocking was enforced before *E. cisalpinus* was recognised as a species distinct from *E. lucius*. Therefore, the use of non-local breeders originating from Central and Northern Europe to produce large numbers of hatchery-reared juveniles has been unintentionally introducing northern pike into Italian freshwater habitats for decades [22]. The sympatric presence of formerly isolated species likely resulted in the breakdown of reproductive barriers between *E. cisalpinus* and *E. lucius* [15,18]. Consistent with this picture, introgressive hybridisation was observed in many populations along the drainage basins in Northern Italy: overall, nearly 20% of the surveyed individuals were late-generation hybrids [18]. This finding indicated that F1 (first filial generation) hybrids were viable and fertile, albeit their rarity suggested they were not favoured over pure southern pike [18]. It is likely that the interplay between the small population sizes of native individuals and large numbers of released non-native individuals counterbalanced the reduced reproductive success of hybrids, thus facilitating their persistence and spread in the wild [23]. Therefore, ongoing introgressive hybridisation between the two species is an issue that may jeopardise the success of conservation and management actions aimed at halting the decline of southern pike, as the remaining pure populations are scant and isolated [24].

The picture is worsened by the use of pike for recreational purposes as they are viewed as high-prize game fish [25]. Indeed, both esocids were extensively bred and introduced in Southern and Insular Italy, where the autochthonous Italian species, *E. cisalpinus*, was also not native [26]. These often illegally introduced populations represent a potential threat to the native aquatic animal community; nevertheless, they also represent an opportunity to understand population dynamics that can also occur in areas where the species is native. In this context, the present study is focused on a pike population that has been recently introduced in an artificial basin, Alto Flumendosa Lake [27], which is located in Eastern Sardinia (in the Western Mediterranean). Overall, the autochthonous freshwater ichthyofauna of Sardinia are limited to very few species [28], whereas dozens of alien fish are currently present on the island, whose introduction has been mostly perpetrated during the past three decades, together with other alien aquatic species [5,27,28]. Alto Flumendosa Lake accurately represents this situation, as nearly all its freshwater ichthyofauna are allochthonous [27,28,29,30,31], aside from *Salariopsis fluviatilis* [30]. 

Information on the introduction of pike in Sardinia is scant, but it should date back more than a decade to when anglers started to report their presence. Moreover, both captivity-born juveniles and wild adult breeders might have been introduced on several occasions, as catches included both small and large pike (AN, *pers. comm*.). Therefore, the current population could have been founded by a mixture of several cohorts and wild adult individuals. In this context, the gathering of information on this population is fundamental as genetic data may help address issues regarding its introduction, origin, and genetic makeup that were only partly unravelled by the previous study [27]. Indeed, mitochondrial DNA markers (mtDNA) grouped all individuals from Alto Flumendosa Lake into the *E. cisalpinus* clade and the overall mtDNA diversity was comparable to that observed in wild southern pike populations from the mainland [27]. However, mtDNA data did not allow for the assessment of whether the population introduced in Alto Flumendosa Lake entirely consisted of pure southern pike or showed introgressive hybridisation with the northern pike, nor did it enable the determination of its likely origin.

To fill this gap, the present study investigated patterns of genetic variation in southern pike that were introduced in Sardinia using the same set of microsatellite markers employed by Gandolfi et al. [18]. The main goals of this study were as follows: (1) to gather the baseline genetic information that may pave the way to answering questions regarding the introduction, origin, causes of successful establishment, and spreading potential of this population; (2) to ascertain whether this population contains only pure individuals of the *E. cisalpinus* variety or hybrids between the former and *E. lucius* as well. This information may also help understand the evolutionary trajectory of this allochthonous southern pike population, which can then be used as both a model and test case for the management of wild populations in the species’ native range where hybridisation still occurs.

## 2. Materials and Methods

### 2.1. Study Area

**Figure 1:**
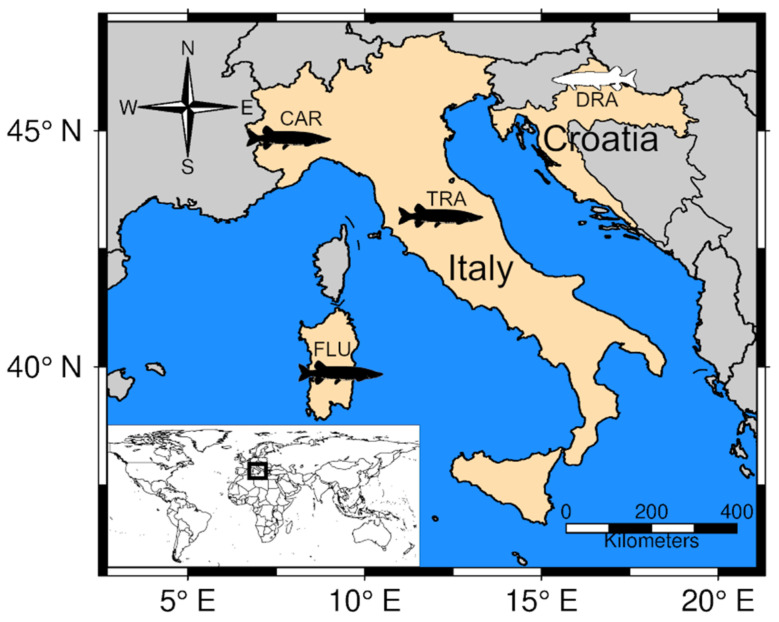
Sampling localities of *Esox cisalpinus* (black) and *E. lucius* (white) populations. FLU: Alto Flumendosa Lake; TRA: Trasimeno Lake; CAR: Carignano-La Loggia; DRA: Drava river. The square in the inset map shows the geographical location of the study area.

Alto Flumendosa Lake is an artificial freshwater basin of about 3.5 km^2^, with an average depth of 20 m, which is located in central Sardinia (Figure 1) at 800 m above sea level. The lake originated as a result of the construction of a dam over the Flumendosa river flowing across the Bau Muggeris valley. 

Currently, the lake harbours mostly allochthonous fish, such as the tench *Tinca tinca* (Linneus, 1758), the rudd *Scardinius erythrophthalmus* (Linnaeus, 1758), the bleak *Alburnus alburnus* (Linnaeus, 1758), the goldfish *Carassius auratus* (Linnaeus, 1758), the European perch (*Perca fluviatilis*), the Italian spined loach (*Cobitis bilineata*), and the roach *Rutilus aula* (Bonaparte, 1841), the latter representing the first observation in Sardinian freshwaters [27]. Moreover, the presence of the brown trout *Salmo trutta* (Linnaeus, 1758) and the rainbow trout *Oncorhynchus mykiss* (Walbaum, 1792) is also reported, as the lake was being actively restocked with both species until a few years ago.

### 2.2. Sampling, DNA Extraction, and PCR Protocols

During 2013–2018, caudal fin-clips were collected from *E. cisalpinus* and *E. lucius* from four localities (Figure 1). Individuals of *E. cisalpinus* variety from Alto Flumendosa Lake (FLU, *N* = 60) were caught with shore-fishing equipment (fishing rod). Individuals of *E. cisalpinus* variety from Carignano-La Loggia (CAR, N = 28) and Trasimeno Lake (TRA, N = 20) as well *E. lucius* from Drava river (DRA, N = 22) were caught by electrofishing (see Casu et al. [27] for further details). Those individuals were then subjected to a non-lethal sampling method by means of small tissue portion removal (fin-clips) and immediately transferred to a recovery tank before being released. Caudal fin-clips were placed in 1.5 mL test tubes filled with 96% ethanol and carried to the laboratory, where they were stored at −20 °C until DNA isolation. Genomic DNA was purified using the Macherey-Nagel NucleoSpin Tissue kit according to the supplier’s instructions and the concentration was estimated by spectrophotometry at 260 nm using a Nanodrop Lite Spectrophotometer (Thermofisher Scientific).

A set of 16 microsatellite markers was amplified using four multiplex Polymerase Chain reaction (PCR) procedures, which were performed in accordance with the protocols outlined by Gandolfi et al. [18]. Reactions were carried out in a total volume of 10 µL. On average, 20 ng of total genomic DNA was combined with 0.06–0.14 µM of each primer and one pellet of PuReTaq Ready-To-Go PCR beads (GE Healthcare, Wauwatosa, WI, USA) containing stabilisers, bovine serum albumin (BSA), deoxynucleotide triphosphates, 2.5 units of PuReTaq DNA polymerase, and reaction buffer. When a bead was reconstituted to a 10 µL final volume, the concentration of each dNTP and MgCl_2_ was set at 200 µM and 1.5 mM, respectively. PCRs were performed in a Bioer LifePro Thermal Cycler under the following conditions: an initial denaturation step at 94 °C for 2 min; 35 cycles at 94 °C for 20 s,52, 54 or 60 °C depending on the multiplex for 10 s and 65 °C for 0.40 s; a final extension at 65 °C for 10 min. After checking for successful amplicons by electrophoresis on a 2% Agarose gel stained with Gel Red Nucleic Acid Stain (Biotium Inc., Fremont, CA, USA), 1 μL of PCR products mixed with 9.90 μL of Formamide and 0.10 μL of GeneScan 500(-250) LIZ size standard (Applied Biosystems, Foster City, CA, USA) was run on an ABI PRISM 3730xl Genetic Analyser (Applied Biosystems) by an external service (GenoScreen, Lille, FRA). Microsatellite alleles were scored and binned using STRand 2.4.110 analysis software [32].

### 2.3. Marker Validation

Departures from Hardy–Weinberg proportions (HWP) and linkage disequilibrium (LD) were tested as suggested by Waples [33]. First, the probability of HWP departures for either heterozygote deficit or heterozygote excess and LD were computed using the Markov chain method (10,000 dememorization steps; 100 batches of 10,000 iterations each) implemented in GENEPOP 4.7 [34]. Then, the cumulative binomial distribution was used to assess whether the number of significant tests for either heterozygote deficit or excess was larger than that expected by chance (alfa = 0.05). In this case, multiple Comparison Procedures (MCP) based on the False Discovery Rate (FDR) were used to adjust the probability values of single tests using the B-Y method [35]. Tests were grouped by population for HWP and by locus pair for LD as recommended by Waples [33], and the FDR correction was applied to each of these groups separately to minimise type II errors. MCP procedures were automated by running a script in R 4.0.2 statistical environment [36], which was used by Cossu et al. [37] and is available on DRYAD (https://doi.org/10.5061/dryad.dm97908).

The microsatellite dataset was corrected for the presence of null alleles, allelic dropout, and other genotyping errors using the Maximum Likelihood approach implemented in MICRODROP 1.01 [38]. The method does not assume HWP to estimate the frequency of genotyping errors; therefore, it can distinguish between actual homozygosity excesses and null alleles under potential inbreeding.

Two methods were used to detect outlier loci. BayeScan 2.1 [39] compares a neutral model with a model that include selection. Setting higher prior odds for the former (threshold = 10), proposal distributions were adjusted using 20 pilot runs of 5000 iterations each, and then a simulation was run for 150,000 iterations. Records were sampled every 20 iterations after discarding the first 50,000 iterations. The FDR (False Discovery Rate) was set at 5% to correct for multiple testing. The second method is implemented in ARLEQUIN 3.5.2 [40] and is based on the FDIST2 approach with a finite island model of migration [41]. The model was run assuming 100 demes and 100,000 permutations. Probability values were corrected using the B-Y method. Only loci detected as outliers by both methods were deemed as true candidates for selection [42].

### 2.4. Patterns of Genetic Variation

Within population summary statistics, population size reductions and contemporary effective population sizes were assessed following Cossu et al. [37]. The number of alleles (*N*_A_), the allelic richness (*A*_R_), the expected and the observed heterozygosity (*H*_E_ and *H*_O_), and the inbreeding coefficient (*F*_IS_) were computed using diveRsity 1.9.9 [43]. The contemporary effective population size (*N*_e_) was estimated using the linkage disequilibrium (LD) method [44,45,46] implemented in NeEstimator V2.1 [47], setting the option to remove singleton alleles (i.e., alleles that occur in in a single heterozygote).

Global and population pairwise genetic differentiation was estimated using diveRsity to compute the Weir and Cockerham’s *F*_ST_ estimator *θ* (1984) [48] and Jost’s *D*_EST_ [49] indexes; means and confidence intervals were assessed carrying out 10,000 bootstrap replicates. Exact G tests implemented in GENEPOP 4.7 were used to compute the probability values regarding population differentiation. Whenever necessary, MCP were used to adjust the probability values by means of the B-Y method [35].

Genetic structure was investigated using Bayesian model-based clustering implemented in STRUCTURE 2.3.4 [50]. Only genetic information was used to group individuals into clusters that minimise Hardy–Weinberg-Linkage disequilibria. Simulations were run using the admixture model with correlated allelic frequencies [51] and varying number of clusters (*K* = 1–7). For each *K*, 10 independent runs were performed, each consisting of 100,000 iterations following a burn-in period of equal length. Two methods were used to detect the most likely number of clusters in the data: the Δ*K* statistics [52], which retrieves the uppermost hierarchical structure, and the method of Puechmaille [53], which addresses issues related to unbalanced sampling design. Both statistics were computed using the pipeline available in STRUCTURESELECTOR [54], which was also used to merge replicate runs from the best *K*, if any, by means of CLUMPAK [55]. The R package POPHELPER 2.3.1 [56] was then used to plot the output.

In addition to model-based Bayesian clustering, Discriminant Analysis of Principal Components (DAPC), which does not rely upon a population genetic model [57], was carried out in the ADEGENET 2.1.5 R package [58]. DAPC was carried out using either sampling populations as prior groups or the function find.clusters() to detect the most likely number of a priori groups. The detection of groups was automated using the method diffNgroup and the Bayesian Information Criterion (BIC). Finally, the number of retained principal components (PC) was optimised using a cross-validation procedure to avoid overfitting of the data.

### 2.5. Hybrid Detection

Two alternative methods, which are specifically tailored to detecting hybrids by assigning individuals to a given genotypic class, were used. These methods, implemented in NEWHYBRIDS 1.1 beta [59] and SNAPCLUST [60], are complementary; although both perform comparably well, the former tends to retrieve pure individuals more accurately whilst the latter detects hybrids, especially late-generation ones, more easily [60]. NEWHYBRIDS uses Bayesian model-based clustering with a Markov Chain Monte Carlo (MCMC) algorithm to compute the posterior probabilities of assigning individuals to pure or hybrid classes. Five replicate runs of 500,000 iterations after a burn in of 100,000 were carried out assuming Jeffrey’s flat priors. SNAPCLUST is implemented in the R package ADEGENET and combines a geometric approach with fast likelihood optimisation based on the Expectation–maximisation (EM) algorithm to identify hybrids between the two parental populations.

Although Gandolfi et al. [18] showed that the microsatellite panel used here can accurately detect hybrids, case-specific simulations were carried out to test the power of both algorithms to detect hybrids in our dataset. Indeed, the reliability of assignment methods depends not only on the number of markers but also on the hybridisation rate and the sampling quality [61]. In the present study, the reference populations used for *E. cisalpinus* (TRA) and *E. lucius* (DRA) could not be the parental populations of any hybrid individual. Therefore, simulations mimicked realistic scenarios of northern pike genomic introgression into southern pike populations that aimed to determine how sampling might affect the assignment of individuals to hybrid classes. In the first scenario (S1), the two reference populations were used to simulate hybridisation in a hatchery between *E. lucius* and *E. cisalpinus*; both northern and southern pike from Europe and Trasimeno Lake, respectively, are used for restocking purposes in hatcheries [18]. The function hybridise(), which is implemented in the R package ADEGENET, was used to create 100 individuals for each of the following classes: pure southern pike, F2, and first and second generation backcrosses between southern pike and hybrid individuals (*N* = 500 individuals). Gametes were sampled with replacement from a multinomial distribution from the given allele frequencies of reference populations. Then, 50 individuals were randomly sampled to create a population that was a mixture of both pure and hybrid southern pike, which was analysed together with the original reference populations. In the second scenario (S2), the evolution of ideal microsatellite markers was simulated in four populations using EASYPOP 2.01 [62]. A hierarchical finite island model was used to mimic levels of genetic variation within and among populations comparable to those that were observed in our dataset; accordingly, two demes (A and B) each consisting of two populations (A1, A2, B1, and B2, respectively) were created. Two simulated populations (A2 and B1), one per deme, were used as baselines to replicate the simulation of both hybrids and an introgressed population (A2B1) as outlined above for the real microsatellite data. Then, a dataset containing A2B1, A1, and B2 populations was assembled to assess how reference but not parental pure populations affect the detection of hybrids in a mixed population.

## 3. Results

### 3.1. Marker Validation

All microsatellite loci, except Elu2, were polymorphic at the 5% level across populations, with the number of alleles ranging from 4 to 33 (Supporting information, Table S1). Among the tests departing from the HWP (*p* < 0.05), those showing an excessive number of heterozygotes (3 out 63) did not exceed the number of tests expected by chance based on the cumulative binomial distribution (5, *p* < 0.05). In contrast, the HWP departures due to a heterozygote deficit exceeded these expectations (25 out 63 tests). Overall, 12 out of 16 loci departed from the HWP in at least one population, which decreased to 9 after applying FDR correction for multiple testing (14 out 63 tests). HWP departures were only found in *E. cisalpinus* populations, with Alto Flumendosa Lake (FLU), Trasimeno Lake (TRA), and Carignano-La Loggia (CAR) showing heterozygote deficits at seven, five, and two loci, respectively.

Overall, 78 out of 480 locus pairs showed LD, which exceeded those expected by chance, based on a cumulative binomial distribution (31 locus pairs, *p* < 0.05). After applying FDR correction, the number of locus pairs showing LD decreased to 17; the number of locus pairs at LD per population ranged between one (TRA) and seven (CAR), with only the locus pair EmaB120-Elu19 showing LD in two populations (FLU and TRA). However, as previous studies did not evidence LD between these loci (Gandolfi et al. 2017), we deemed it unlikely that LD was due to a truly non-random association.

Overall, rates of allelic dropout (null alleles and other genotyping errors) were low at most loci, ranging between 0 and 0.08, except for five loci, namely, B457, B422, Eluc014, and Elu51, which showed rates ranging between 0.15 (B457) and 0.39 (Elu51). A slight correlation between the number of missing data and homozygotes was observed across individuals (Pearson’s *r* = 0.034, *p* = 0.43); therefore, the dataset was corrected for allelic dropout and 5 out 16 microsatellite loci were not considered for downstream analyses as they were either monomorphic (Elu2) or showed >12% missing data in at least one population (B422, Eluc014, EluB38bis, and Elu51).

No locus was detected as a potential candidate for selection by both outlier detection methods simultaneously. The Bayesian method did not find any potential outlier, whereas the FDIST2 approach indicated Eluc040 as a potential candidate for divergent selection (Supporting Information, Figures S1 and S2). Therefore, the loci were putatively neutral after analysis.

The following sub-sections show the results based on the dataset that was corrected for genotyping errors. This dataset, together with the raw data (which were not corrected for genotype errors), input data for simulations, R scripts for running the analyses, and a comparison of outcomes based on both the corrected and uncorrected genetic data are available on figshare repository (https://doi.org/10.6084/m9.figshare.20382297.v1). 

### 3.2. Patterns of Genetic Variation

Mean and per-locus summary statistics depicting genetic diversity within populations are shown in Table 1 and in Supporting Information—Table S2, respectively. 

The highest values of expected (*H*_E_) and observed (*H*_O_) heterozygosity were found in the population from Alto Flumendosa Lake (referred to as FLU hereafter). In contrast to the northern pike population, all southern pike populations showed an excessive number of heterozygotes (*H*_O_ > *H*_E_; Table 1). However, only the population from Trasimeno Lake (TRA hereafter) showed a negative inbreeding coefficient (*F*_IS_ = −0.217) with a 95% confidence interval (CI_95_ hereafter) that did not span zero. The southern pike populations also displayed smaller mean allelic richness (*A*_R_) than the northern pike population from the Drava river (DRA hereafter), with the smallest value was recorded at TRA (*A*_R_ = 2.90 ± 0.40). Mean estimates of the contemporary populations’ effective size (*N*_e_) as well as the CI_95_ were finite (Table 2) when full samples were used. 

FLU showed the largest effective size (*N*_e_ = 66, CI_95_ = 36–189) and TRA the smallest (*N*_e_ = 6, CI_95_ = 2–23). However, after setting *S* = 20 for all populations, the *N*_e_ estimates were very similar across all populations (Table 2).

Hereafter, only results based on *F*_ST_ are reported (Table 3), as those based on the *D*_EST_ values showed the same trend (Supporting Information, Table S3). The global *F*_ST_ showed a significant degree of genetic differentiation amongst the populations (*F*_ST_ = 0.338; CI_95_ = 0.315, 0.361), with heterogeneous genotypic frequencies amongst samples evidenced by Fisher’s exact test of population differentiation (*χ*^2^ = 350.732 with 22 degrees of freedom; *p* < 0.001). 

Both the pairwise *F*_ST_ values and exact G tests after correction for multiple testing (FDR method) evidenced genetic divergence amongst all population pairs (Table 3). As expected, the largest degree of genetic divergence was observed between the northern and southern pike populations, except one noteworthy case. Indeed, the degree of genetic divergence between Carignano-La Loggia (CAR hereafter) and TRA was larger than that between FLU and DRA (*F*_ST_ = 0.365 and 0.320, respectively), albeit the CI_95_ slightly overlapped.

**Figure 2:**
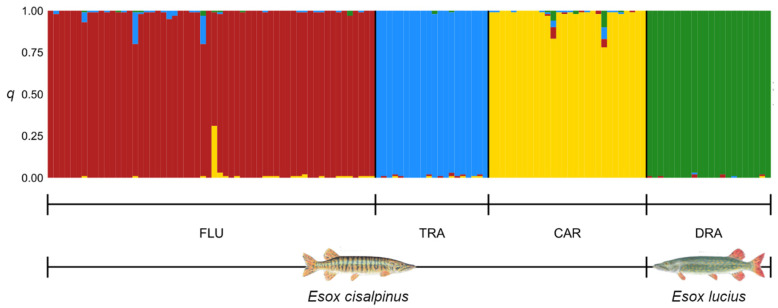
Most likely genetic structure inferred by Bayesian model-based clustering. The model with *K* = 4 (*K* = number of genetic clusters) was selected out of models in which *K* ranged from 1 to 7 by both Evanno et al.’s and Puechmaille’s methods [52,53]. Each individual is represented by a thin vertical bar, which is partitioned into *K*-coloured segments. The height of each segment is proportional to the individual membership coefficient (*q*) in the corresponding cluster. Populations are abbreviated as in Figure 1 and Table 1.

Both Δ*K* and Puechmaille’s statistics clearly supported *K* = 4 as the best Bayesian-clustering solution (Supporting information, Figure S3). The genetic clusters tightly fit the sampling localities (Figure 2) and 96% of individuals were assigned to a single cluster with high membership coefficients (*q* > 0.90). 

Only five individuals (three from FLU and two from CAR) were admixed (*q* < 0.80); among these individuals, only those from CAR showed a small membership (0.06 ≤ *q* ≤ 0.10) to the cluster in which all northern pike were assigned (Figure 2).

**Figure 3:**
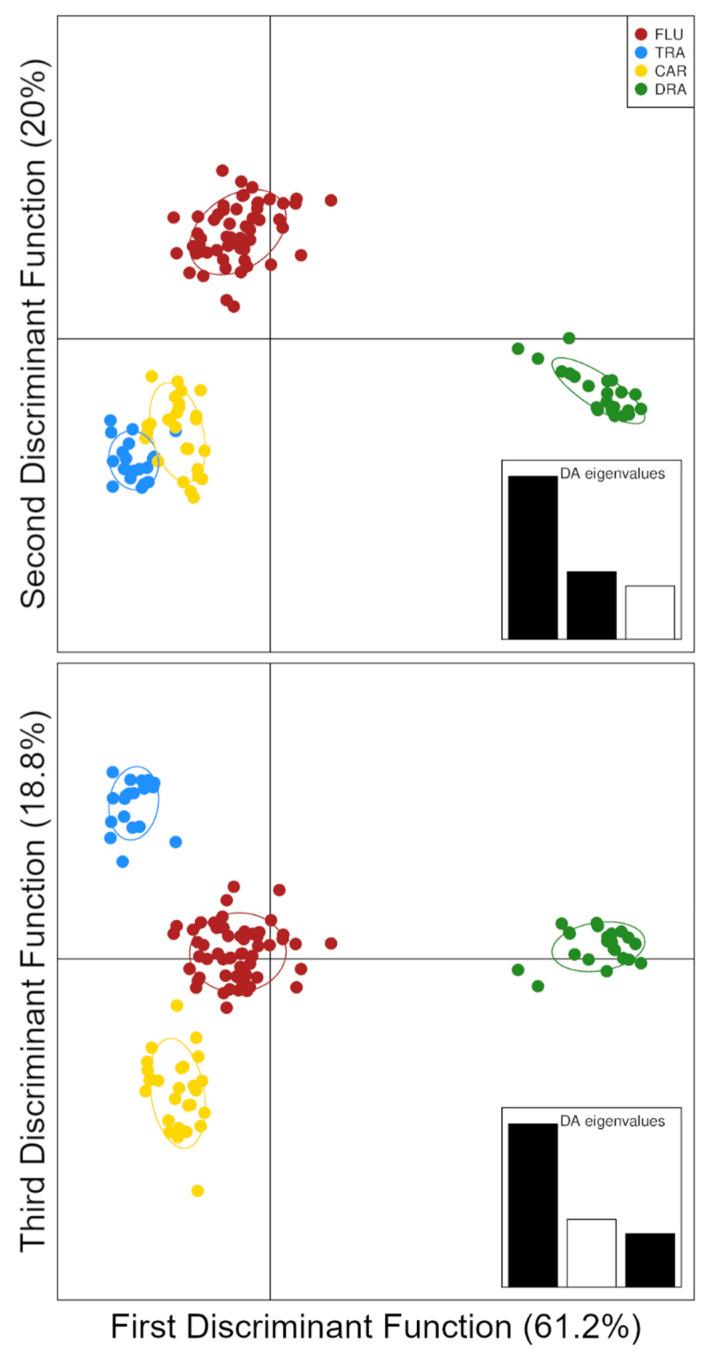
Genetic structure inferred from Discriminant Analysis of Principal Components (DAPC). Each dot represents an individual fish and is coloured according to the sampling locality as well as the 95% inertia ellipses. Black bars in each inset show which discriminant functions are plotted. Bar height is proportional to the amount of genetic variation explained by each DF.

The outcomes of the Discriminant Analysis of Principal Components (DAPC) closely mirrored those obtained by Bayesian clustering, regardless of whether the sampling localities (Figure 3) or K-Means inferred clusters (Supporting information, Figure S4) were used as prior groups.

Since the inferred clusters perfectly matched the sampling localities (Supporting information, Figure S4), only the DAPC results based on the latter are depicted and discussed henceforth. Discriminant analysis was carried out, retaining the first 30 principal components selected by the cross-validation procedure. The ordination plots of the discriminant functions (DF) highlighted the hierarchical genetic structure of the data (Figure 3). The first DF, which accounted for most of the genetic variation, highlighted the separation between the two species, *E. lucius* and *E. cisalpinus*, whereas the remaining DFs showed the genetic structure within the latter. The second DF separated FLU from the mainland populations, whilst the third DF highlighted the differences between CAR and TRA.

### 3.3. Hybrid Detection

**Figure 4:**
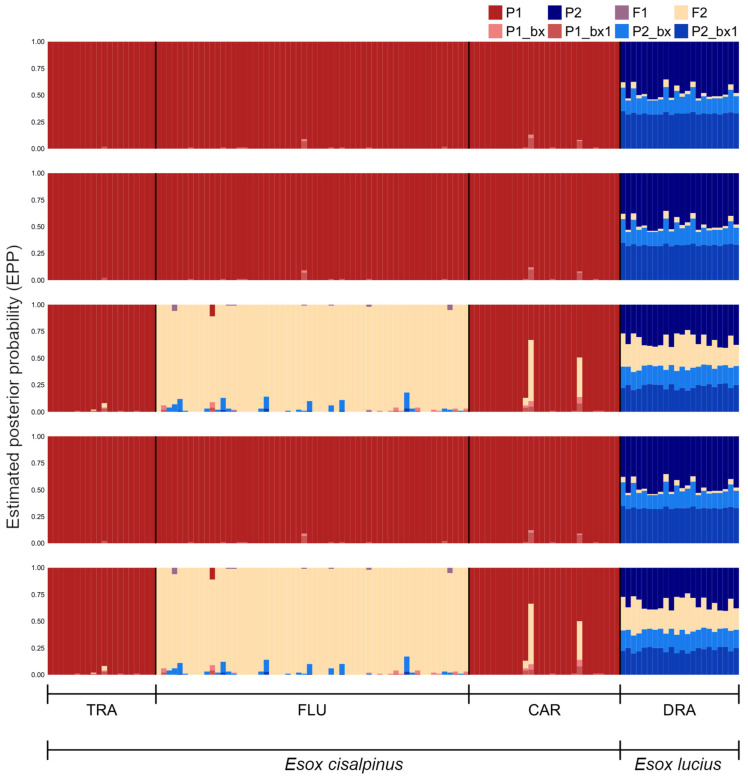
Bayesian model-based assignment of individuals to different genotypic classes. Each bar plot represents the output of an independent run of the algorithm, in which individuals are represented by vertical bars subdivided into coloured segments. The height of each segment corresponds to the assignment probability of an individual to a given genotypic class. P1 and P2 represent pure parental classes. Other classes correspond to F1 and F2 hybrids, that is, first- and second-generation backcrosses between F1 with southern (P1_bx and P1_bx1, respectively) and northern pike (P2_bx and P2_bx1, respectively).

The Bayesian algorithm implemented in NEWHYBRIDS did not show consistent results across the five replicate runs (Figure 4). 

In three out of five runs, nearly all individuals from TRA (the reference population for *E. cisalpinus*), FLU, and CAR were classified as pure with high posterior probabilities (*q* > 0.9) and assigned to the same genotypic class (Parental population 1, P1). In contrast, in 2 out of 5 runs, all individuals from FLU and one from CAR were classified as hybrids and assigned to the F2 genotypic class with *q* > 0.50; however, 51 out of 58 individuals from FLU were classified as F2 hybrids when *q* > 0.90 was used as the criterion to assign individuals to a given genotypic class. In contrast, more than half of the individuals from DRA (the reference population for *E. lucius*) could not be assigned to any genotypic class, even when adopting a more liberal threshold (*q* > 0.50). In three out of five runs, ten individuals were classified as pure and assigned to the parental population P2 using this threshold. Only ten individuals from DRA (the reference population for *E. lucius*) were classified as pure and assigned to the other parental category (P2) using the most liberal threshold (*q* > 0.50) in three out of five runs. Otherwise, they were left unassigned across all runs if more conservative criteria were used (*q* > 0.75) and even when the most liberal threshold was adopted in two out of five runs.

**Figure 5:**
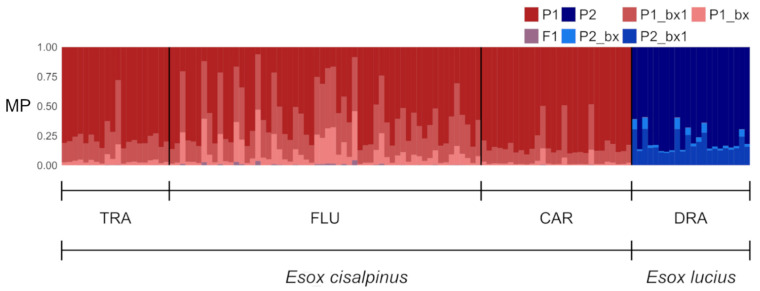
Assignment of individuals to different genotypic classes based on the Expectation–Maximisation algorithm. The bar plot shows the membership probability (MP) of each individual of being a pure southern or northern pike, an F1, or a backcross hybrid between the two parental species. Genotypic classes are arranged as in Figure 4; note, however, that the algorithm implemented in SNAPCLUST does not allow one to distinguish F2 hybrids.

The EM algorithm implemented in SNAPCLUST assigned most of the individuals (81%) from TRA, FLU, and CAR to the parental population P1 under the most liberal threshold (Figure 5). 

At *q* > 0.75, the fraction of individuals assigned to P1 decreased to 55%, and not even a single individual was assigned when the most conservative threshold was adopted. Overall, 7 out of 106 southern pike were classified as second-generation backcross hybrids: one individual from TRA and six from FLU showed assignment posterior probabilities barely exceeding 50% for this genotypic class. All individuals from DRA were assigned to the other parental population, P2, when *q* > 0.50 (Figure 5). The fraction of individuals that was successfully assigned to P2 decreased to 73% as the threshold increased and dropped to zero when the most conservative threshold was enforced.

In contrast to the observed data, S1 showed consistent results within and across both methods (Supporting Information, Figures S5–S8). Overall, NEWHYBRIDS (Supporting information, Figures S5 and S6) showed larger posterior assignment probabilities than SNAPCLUST (Supporting Information, Figures S7 and S8). The latter, however, assigned individuals from DRA to the parental population more effectively than the former (22 and 5 out of 22 individuals, respectively at *q* > 0.5). SNAPCLUST also successfully assigned most of the individuals from DRA at *q* > 0.75 (18 out of 22 individuals). Both methods detected hybrids only when the most liberal threshold was enforced, except for three out four F2 hybrids, which were detected by NEWHYBRIDS even at *q* > 0.9. Parental individuals were not misclassified as hybrids (false positives), whereas 21% of hybrids were assigned to a parental class (false negatives). SNAPCLUST detected backcrosses slightly more effectively than NEWHYBRIDS (47% and 37%, respectively).

The trends outlined above resembled S2, even though both methods performed slightly better than S1 (Supporting Information, Figures S9–S12). All parental individuals were correctly assigned with higher posterior probabilities than in S1, and NEWHYBRIDS (Supporting Information, Figures S9 and S10) overall showed larger assignment probabilities than SNAPCLUST (Supporting Information, Figures S11 and S12). Both methods also showed lower false negative rates than S1 (17% and 8% for NEWHYBRIDS and SNAPCLUST, respectively). The latter method also provided more accurate detection than the former backcrossed hybrid individuals (58% and 42%, respectively). Overall, disregarding the exact hybrid class to which simulated genotypes were assigned, the detection rate was roughly the same in S1 (73%), whereas SNAPCLUST performed slightly better than NEWHYBRIDS in S2 (92% and 83%, respectively).

## 4. Discussion

The present study highlights the fact that the genetic diversity and effective population size of the population of *Esox cisalpinus* introduced in Alto Flumendosa Lake are comparable to those of wild populations living in closed freshwater habitats without signs of a founder effect. This population does not entirely consist of pure southern pike, as the presence of late-generation hybrids cannot be completely ruled out. Our results exclude Trasimeno Lake as a potential source population and hence complement those of our previous study [27], which evidenced that populations from Alto Flumendosa Lake and Northern Italy have similar degrees of mtDNA variation. Determining its presence outside native range areas would require further genetic studies; nonetheless, we suggest that this population deserves to be constantly monitored as it could be used as a model to understand the evolutionary trajectory of isolated populations and thus as a test case to ascertain the management outcomes on wild populations in the species’ native range where hybridisation is still present.

### 4.1. Marker Validation

HWP departures are expected in fish populations and may be expected in those characterised by small sample sizes and limited gene flow such as southern and northern pike [12]. For instance, the cited authors found that pike populations inhabiting small and completely isolated lakes departed from the HWP. However, most departures should consist of an excess rather than a heterozygote deficit if small population sizes are the main driver of HWP departures [33]. Instead, in our dataset, nearly all departures depended on a heterozygote deficit, which likely stems from genotyping errors such as null alleles and allelic dropouts. Indeed, the loci characterised by a heterozygote deficit decreased and *F*_IS_ 95% confidence intervals encompassed zero in three out of four populations (FLU, CAR, and DRA) after genotyping errors were corrected (Table S2). Therefore, the sections below illustrate the results based on the corrected dataset, albeit the use of raw data barely affects the outcomes of genetic analyses (figshare, DOI: 10.6084/m9.figshare.20382297.v1).

### 4.2. Overall Patterns of Genetic Diversity

The populations analysed in the present study show low levels of genetic diversity (Table 1) and large population differentiation (Table 2), which are comparable with the genetic patterns observed in most studies on southern and northern pike (e.g., Lucentini et al. [16,17], Bekkevold et al. [19], and Gandolfi et al. [18]). Both historical and contemporary factors have been invoked to explain such patterns in European populations. For instance, low levels of genetic variation in freshwater top predators such as pike may stem from limited gene flow and small population sizes as a result of low-density populations inhabiting closed and potentially isolated habitats [17,19]. Consistent with this picture, all the populations investigated in the present study show small effective population sizes (Table 3), whose magnitudes are comparable to those reported in pike populations living in closed freshwater habitats [3,17,63].

Remarkably, TRA shows the smallest effective population size (*N*_e_ = 6, CI_95_ = 2–23), albeit this population has been undergoing supportive breeding since 1970 [17]. Our *N*_e_ estimate, which is twice to five-fold smaller than those obtained over different time periods by Lucentini et al. [17], might be downwardly biased by the small sample size of TRA (*S* = 20). Indeed, *N*_e_ estimators tend to underestimate effective population size when the sample size ratio corresponding to the true effective population size (*S*/*N*_e_) is too small [64]. For instance, FLU shows a five-fold decrease in effective population size when setting *S* = 20 to estimate *N*_e_ (Table 3); assuming a similar proportional decrease for TRA, *N*_e_ would be as large as the previously obtained estimates [17]. Regardless of the sample size, all populations show finite estimates, which fits well the picture of a small true *N*_e_ in southern pike populations. Indeed, *N*_e_ estimators tend to be more reliable and precise when the true effective population size is <500 individuals; when it exceeds this threshold or *S*/*N*_e_ < 0.1, the LD method can yield infinite estimates of *N*_e_ [45,46].

All the methods used to investigate genetic structuring show concordant results, regardless of whether they were grounded on explicit genetic models such as Bayesian clustering (Figure 2) or not such as DAPC (Figure 3). Moreover, when only genetic information is used to group individuals, the retrieved clusters tightly match the sampling localities, thus suggesting the presence of genetically divergent populations with little to no admixture (Figure 3). In contrast to Gandolfi et al. [18], the Bayesian clustering method used in this study did not find clusters corresponding to the species *E. lucius* and *E. cisalpinus* when *K* = 2 (Supporting information, Figure S3). The inconsistent outcomes observed at *K* = 2 likely depend on the high degree of genetic divergence between populations of *E. cisalpinus* considered in the present study, which may determine strong HWP departures and linkage disequilibrium within southern pike. Accordingly, DAPC, which does not rely upon an explicit genetic model, showed a pattern in which the first discriminant function separates the two species, whilst the others depict the genetic variation within *E. cisalpinus* (Figure 3).

### 4.3. Hybrid Detection

In the present study, notwithstanding Bayesian clustering points to pure populations, the presence of late-generation hybrids cannot be completely ruled out. The two algorithms, which are specifically tailored to detecting hybrids, yield contrasting and uncertain results, as discrepancies occur between and within methods (Figure 4 and Figure 5). Such uncertainty does not depend on the microsatellite loci, which showed almost perfect capacity for assigning the simulated individuals to the correct genotypic class in a previous study [18]. Moreover, both hybrid detection methods perform well and consistently when either microsatellite data obtained in the present study (Supporting information, Figures S5–S8) or ideal microsatellite loci (Supporting Information, Figures S9–S12) are used to simulate populations that are introgressed by late-generation hybrids. Although a non-negligible fraction of hybrids was misclassified as pure individuals, both methods never misplace pure individuals into hybrid categories. These results agree with the outcomes of Beugin et al. [60], whose extensive simulations showed that the two methods perform comparably well. It should be noted, however, that these results are limited to second-generation backcrosses, as microsatellites have limited power with respect to detecting older backcross hybrid classes [65]. The occurrence of relatively old hybridisation events (beyond the third generation) cannot be ruled out in many wild pike populations, whose genetic structure might have been altered by more than 100 years of extensive stocking and transplantations across Europe [20].

Thus, this scenario may explain the uncertain classification of individuals into genotypic classes that was observed in the present study as, for instance, the incoherent assignment of individuals to pure southern pike or F2 hybrid classes by the Bayesian algorithm (Figure 4). A similar behaviour of NEWHYBRIDS has been reported in wild populations of brown trout stocked with hatchery-reared individuals: hybridisation beyond the second generation resulted in the classification of individuals as either purebreds or F2 hybrids [66]. Alternatively, the contrasting outcomes of NEWHYBRIDS may depend on the violation of the underlying genetic model, which assumes that parental wild populations fulfil HWLE and uses a linkage disequilibrium to distinguish between pure and hybrid individuals [59]. FLU may not fit this picture, as wild breeders might not have randomly selected nor randomly crossed for reproduction in captivity; moreover, they might also come from a wild population that was already a mixture of wild and hybrid pike and that departed from HWLE. Multiple introduction events involving both genetically divergent cohorts and wild adult breeders may also have contributed to fostering departures from HWP and LE. Altogether, these factors may confound the signal that NEWHYBRIDS searches for, leading to unpredictable results, as the assumption that disequilibrium arises only as a result of the mixture of parental and hybrid individuals is violated [59].

A cautionary approach suggests that the potential presence of southern pike introgressed by the northern pike genome cannot be discarded in Alto Flumendosa Lake. Although several studies indicate that NEWHYBRIDS assigns individuals more accurately than other methods [61,65], our simulations highlight the opposite picture: the EM algorithm seems to detect backcrosses slightly better than the Bayesian method, which agrees with the results portrayed by Beugin et al. [60]. Moreover, even though both methods do not detect false negatives in the simulated scenarios, caution suggests that their presence should not be excluded in the real data. For instance, one individual from TRA is classified as a backcross by the EM algorithm (Figure 5), albeit this population should entirely consist of pure southern pike (however, see Bianco [26]). Nevertheless, it seems unlikely that all the six hybrids detected at FLU are false negatives; rather, the simulated scenarios highlight the fact that it is more likely to misclassifying late-generation hybrids as pure southern pike than *vice versa*.

In contrast to FLU, the population from Carignano-La Loggia (CAR) could entirely consist of pure southern pike, even though caution is needed in drawing such a conclusion, as pure individuals might be the descendants of seldom, historical hybridisation events [9]. The older the event, the more likely late-generation hybrids can be overlooked and misclassified as pure individuals [60,66,67,68]. In these cases, however, it is difficult to decide when an individual should still be viewed as a hybrid or rather a member of the population undergoing introgression [66].

The uncertainty with which individuals from the Drava River (DRA) are classified as pure northern pike or left unassigned deserves some consideration. We may speculate that it might be the footprint of the historical introgression of the southern pike genome into the Danubian lineage [15,20,69]. The extensive stocking of pike in Italian hatcheries has not only resulted in the introduction of northern pike south of the Alps but also in southern pike having been exported to many European countries, including Austria, Slovenia, and Croatia [26]. The current absence of hybrids north of the Alps, as well as the rarity of southern pike-like mtDNA, might reflect the poor survival performances of stocked pike, which could be very low depending on several biological and ecological factors [9,18]. Further research on this topic is needed, albeit old hybridisation events are difficult to ascertain [9].

### 4.4. Genetic Makeup of the Alto Flumendosa Lake Population

Compared to the other southern pike populations analysed in the present study, FLU shows higher levels of genetic diversity (Table 1), whose magnitude is similar to the values reported by Gandolfi et al. [18] for both wild and hatchery populations of *E. cisalpinus*. Based on the levels of mtDNA variation, Casu et al. [27] hypothesised that Alto Flumendosa Lake was stocked with the offspring of a large number of breeders. Microsatellite data support this hypothesis, as the *N*_e_ indicates that this population might have been founded by 66 breeders (CI_95_ = 36–183). Moreover, this is likely a conservative estimate, as it has been estimated by a sample consisting of individuals of different ages; therefore, including individuals with overlapping generations might have introduced a downward bias in *N*_e_ estimates [70,71]. To the best of our knowledge, there are few genetic surveys dealing with the stocking of lakes wherein southern or northern pike were previously absent. For instance, the objective of stocking Trasimeno Lake with hatchery-born individuals is to supplement the local wild population [17]; therefore, *N*_e_ estimates represent the effective population size of the combined wild and hatchery populations [10,72]. The *N_e_* estimates obtained in the present study can be compared with those resulting from the introduction of northern pike in north American lakes [3,63], which show small effective population sizes (few dozens of individuals) even though several thousands of pike were censused. These studies also highlight high levels of genetic diversity even though the *N_e_* could have been small over long time periods. Thus, Miller and Kapuscinsky [63] suggested that the current level of genetic diversity could be the remnant of that harboured in the original founder population. The same picture may also hold for FLU, as the number of breeders might have been large enough to maintain the genetic diversity of the wild population from which the parental individuals were collected. Moreover, high genetic diversity may also depend on the presence of introgressive hybridisation with northern pike and/or multiple introductions of genetically divergent cohorts together with adult breeders into Alto Flumendosa Lake. The latter scenario fits with the size range of southern pike that have been caught in the lake during from 2014 to 2019 (Table S4).

Although more populations should be included to infer the potential origin of FLU, TRA can be excluded as the primary source of breeders for southern pike introduced into Alto Flumendosa Lake; for instance, the lower genetic diversity of the latter is at odds with this scenario. Therefore, we may speculate that the origin of FLU should be searched in hatcheries and/or wild populations of Northern Italy, even though the genetic divergence from CAR (Po drainage basin) is as large as that between FLU and TRA (Table 3). Indeed, such genetic differentiation lies within the range reported among populations from the Po Drainage basin [16]. Furthermore, it should be considered that, in addition to the geographic origin of breeders, genetic drift as a result of a founder effect in the hatcheries might have further boosted the divergence between FLU and the other populations. Accounting for uncertainty in hybrid detection, the proportion of hybrids (10%) is comparable with the rates reported in several populations from Northern Italy [18]. This result provides further evidence for the hypothesis that wild adult southern pike and their offspring may come from hatcheries in Northern Italy, which include both pure and introgressed southern pike.

### 4.5. Fate of an Introduced Population: Should the Pike Go or Should They Stay?

The baseline genetic information gathered herein may help assess the origin, introduction events, and the potential spread of the southern pike population that has been recently introduced to Alto Flumendosa Lake. Integrating these genetic data with those of wild and hatchery populations from Italy may unravel the origin of the invading population. The population from Alto Flumendosa Lake is likely a mixture of pure and hybrid southern pike that are introgressed by the northern pike’s genome. Uncertainty with respect to the assignment of the individuals to pure or hybrid genotype classes, and thus in the estimation of their proportions in the introduced population, may be due to the limited capacity to detect late-generation hybrids [65]. Thus, a more powerful panel of molecular markers is needed to improve the detection of late-generation hybrids. Ascertaining the incidence of introgressive hybridisation may further impact the future management of this population.

Overall, the present study highlights, for the first time, that the introgression of northern into the southern pike genome might be underestimated, albeit caution is needed when drawing such a conclusion. Indeed, this result stems from only two simulated scenarios, yet they are scenarios representing realistic cases that may occur in the wild (see Gandolfi et al. [18]). More thorough and exhaustive studies coupling empirical data and simulations based on realistic scenarios are needed to further investigate this issue. This research could be particularly important for Carignano-La Loggia, which likely harbours a population of pure southern pike. This population, which is close to the locality where *E. cisalpinus* was first described [14,26], may be a further important source for the species’ conservation and management and thus must also be genetically monitored.

## 5. Conclusions

The present study aims to assess the genetic makeup and the occurrence of hybrid individuals in a southern pike population that has been introduced in Alto Flumendosa Lake, Sardinia, outside its native range and expand on the results of a previous study [27]. This population shows high genetic diversity, which is consistent with (1) the large numbers of founding breeders, (2) the incidence of multiple introduction events of genetically divergent hatchery-born cohorts and wild adult individuals, and (3) introgressive hybridisation between southern and northern pike. Notably, the presence of hybrid individuals in Sardinia is uncertain, but the presence of late-generation hybrids cannot be ruled out.

Overall, genetic information might help unravel the origin, number of introduction events, and evolutionary trajectory of an alien fish population in a new habitat [73,74]. In particular, comparison with wild and hatchery populations from the southern pike’s native range may help achieve such a goal and understand the evolution of hybridisation in isolated populations. Moreover, the present study issues a warning regarding the conservation of southern pike in its native range, as introgressive hybridisation between northern and southern pike might be underestimated. Developing a panel of markers that may increase the power of detecting late-generation hybrids may improve the design of southern-pike conservation plans in its native areas of distribution.

## Figures and Tables

**Table 1:** Within-population genetic estimates in *Esox* spp. Summary statistics of within-population genetic variation averaged over loci for each population.

	*N*	*N*_A_ ± SD	*A*_R_ ± SD	*H*_E_ ± SD	*H*_O_ ± SD	*F*_IS_ (95% CI)
FLU	58	4.73 ± 1.68	3.91 ± 1.18	0.580 ± 0.187	0.605 ± 0.194	−0.042 (−0.086, 0.002)
TRA	20	3.30 ± 2.36	2.90 ± 1.79	0.385 ± 0.245	0.478 ± 0.311	−0.217 (−0.346, −0.084)
CAR	28	4.18 ± 2.18	3.59 ± 1.64	0.417 ± 0.205	0.435 ± 0.227	−0.043 (−0.131, 0.034)
DRA	22	5.54 ± 3.56	4.86 ± 3.04	0.481 ± 0.318	0.465 ± 0.319	0.036 (−0.024, 0.089)

Abbreviations: FLU—*Esox cisalpinus* from Alto Flumendosa Lake; TRA—*E. cisalpinus* from Trasimeno Lake; CAR—*E. cisalpinus* from Carignano-La Loggia, Po river drainage basin; DRA—*E. lucius* from Drava river, Danubian drainage basin; *N*—sample size; *N*_A_—number of alleles; *A*_R_—allelic richness; *H*_E_—expected heterozygosity; *H*_O_—observed heterozygosity; *F*_IS_ = inbreeding coefficient; SD—Standard Deviation; 95% CI—95% Confidence Interval.

**Table 2:** Within-population genetic estimates in *Esox* spp. Mean contemporary effective population size estimated using the Linkage Disequilibrium method (*N*_e_^˄^ LD).

	*N_e_*^˄^ LD		
	Harmonic Mean	Lower 95% CI	Upper 95% CI
FLU	66 (12)	36 (4)	189 (48)
TRA	6	2	23
CAR	13 (12)	3 (2)	56 (∞)
DRA	14 (13)	6 (3)	46 (105)

Note: Jack-knifing over loci is used to estimate 95% confidence intervals (CI) of *N*_e_. Values between brackets are estimates of effective population size that are based upon a minimum sample size of 20 individuals, which corresponds to the sample size of TRA. Populations are abbreviated as in Table 1. Abbreviations: *N*_e_ = Effective population size; LD = Linkage Disequilibrium; CI = Confidence Interval.

**Table 3:** Within-population genetic estimates in *Esox* spp. Pairwise population differentiation estimated using Weir and Cockerham’s *ϴ*.

	FLU	TRA	CAR	DRA
FLU	—	(0.203, 0.254)	(0.210, 0.276)	(0.293, 0.348)
TRA	0.227	—	(0.322, 0.409)	(0.421, 0.485)
CAR	0.243	0.365	—	(0.440, 0.519)
DRA	0.320	0.451	0.478	—

Note: Observed values are reported below the diagonal. Lower and upper limits of 95% confidence intervals based on 10,000 bootstraps are reported within brackets above the diagonal. Populations are abbreviated as in Table 1.

## Data Availability

The data presented in this study are openly available in FigShare at https://doi.org/10.6084/m9.figshare.20382297.v1. R-scripts used for data analysis and graphics are available by the corresponding author upon reasonable request.

## Supplementary Materials

The following supporting information can be downloaded at: https://www.mdpi.com/article/10.3390/ani13030380/s1, Figure S1: Detection of outlier loci for selection based on the method implemented in BAYESCAN; Figure S2: Detection of outlier loci for selection based on the FDIST2 method implemented in ARLEQUIN; Figure S3: Bayesian clustering outcomes at K = 2; Figure S4: Discriminant Analysis of Principal Components (DAPC) based on K-means clustering and automated group detection; Figure S5: Bayesian model-based assignment of simulated individuals to different genotypic classes; Figure S6: Multiline bar plot obtained after merging the five independent runs illustrated in Figure S5; Figure S7: Assignment of simulated individuals to different genotypic classes based on the Expectation-Maximisation algorithm; Figure S8: Multiline bar plot of individual assignments illustrated in Figure S7; Figure S9: Bayesian model-based assignment of simulated individuals to different genotypic classes; Figure S10: Multiline bar plot obtained after merging the five independent runs illustrated in Figure S9; Figure S11: Assignment of simulated individuals to different genotypic classes based on the Expectation-Maximisation algorithm; Figure S12: Multiline bar plot of individual assignments illustrated in Figure S11; Table S1: Microsatellite loci used in this study; Table S2: Summary statistics of within-population genetic variation for each locus; Table S3: Pairwise population differentiation estimated using Jost’s *D*_EST_; Table S4: Range size distribution of southern pike caught in Alto Flumendosa Lake.
