# Supplementary material for: Appraising the Genetic Makeup of an Allochthonous Southern Pike Population: An Opportunity to Predict the Evolution of Introgressive Hybridization in Isolated Populations?
Source: Animals (Basel). 2023 Jan 22;13(3):380. doi: 10.3390/ani13030380 (PMC9913590; doi:10.3390/ani13030380)
Supplement: Supplementary file 1 [file animals-13-00380-s001.zip › Figures_S1-S12_SuppInfo.pdf]

# Appraising the Genetic Makeup of a Southern Pike Allochthonous Population: An Opportunity to Predict the Evolution of Introgressive Hybridization in Isolated Populations?

Marco Casu[1], Ilenia Azzena[1, 2], Fabio Scarpa[2], Chiara Locci[1,2], Alessio Niffoi[1], Giovanni B. Delmastro[3], Paolo Lo Conte[4], Antonio Varcasia[1], Stefano Bovero[5], Alessandro Candiotto[6], Daria Sanna[2], Piero Cossu[1]

[1] Department of Veterinary Medicine, University of Sassari, Sassari, Italy

[2]Department of Biomedical Sciences, University of Sassari, Sassari, Italy

[3]Laboratorio di Ittiologia, Museo Civico di Storia Naturale, Carmagnola, Italy

[4]Funzione Specializzata Tutela Fauna e Flora, Città Metropolitana di Torino, Torino, Italy

[5]Independent researcher “Zirichiltaggi” Sardinia Wildlife Conservation NGO, Sassari, Italia

[6]NaturaStaff Hydrobiologist, Mongardino, Italy

Corresponding author:

Piero Cossu

Department of Veterinary Medicine, Via Vienna 2, 07100, Sassari, Italy

Phone: +39 079 2280924

Email: picossu@uniss.it

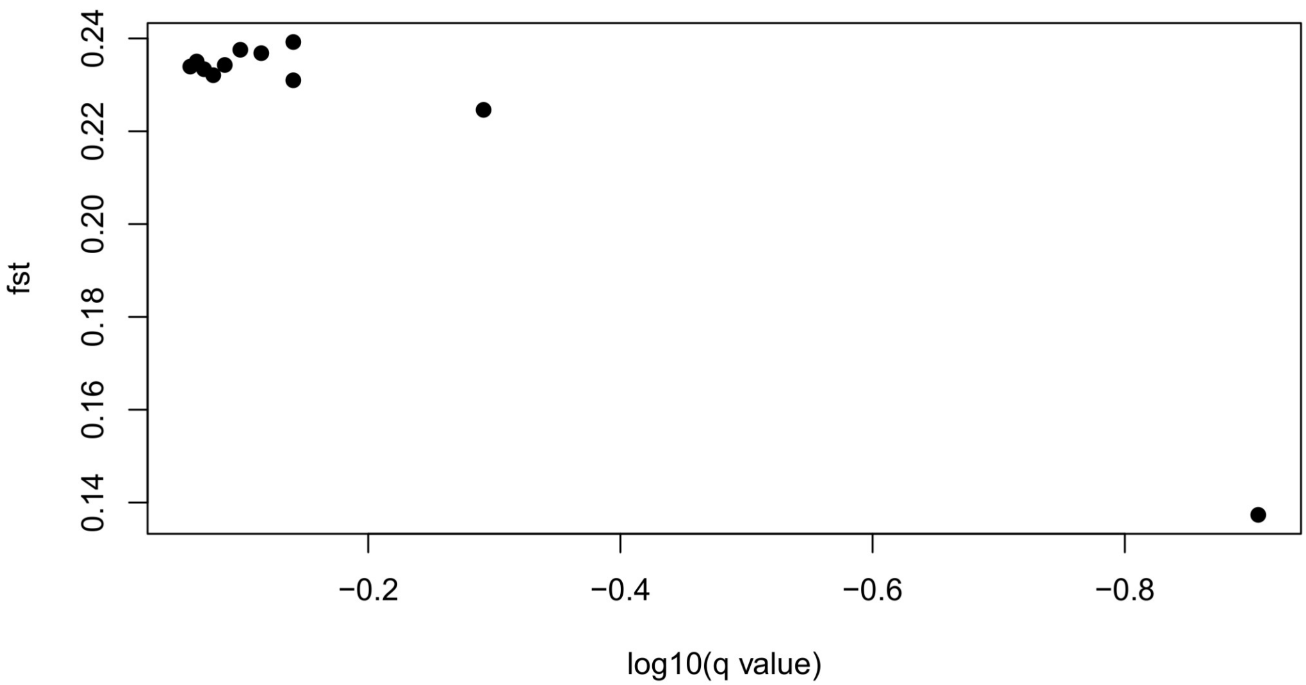


**Figure S1.** Detection of outlier loci for selection based on the method implemented in BAYESCAN. Points represent the $F_{ST}$ values for 11 microsatellite loci, none of which reached the threshold to be detected as an outlier

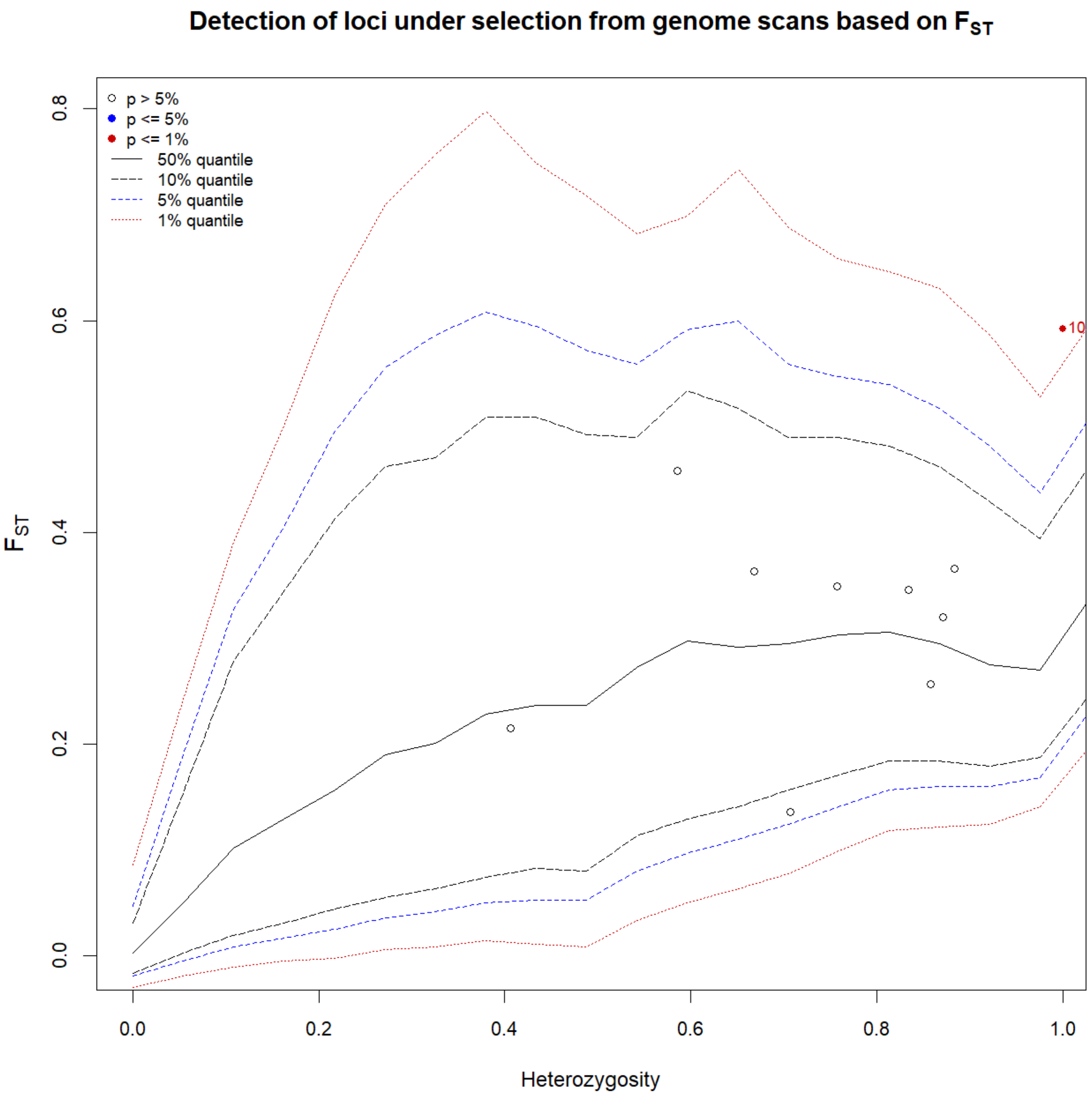


**Figure S2.** Detection of outlier loci for selection based on the FDIST2 method implemented in ARLEQUIN. Circles represent the $F_{ST}$ values for 11 microsatellite loci. Solid and dashed lines outline the quantiles delimiting the distribution of $F_{ST}$ values expected at selective neutrality. Loci are deemed as outlier if their FST values fall above (divergent selection) or below (balancing selection) the expected neutral distribution. The solid red circle marks one candidate locus for divergent selection: Eluc040

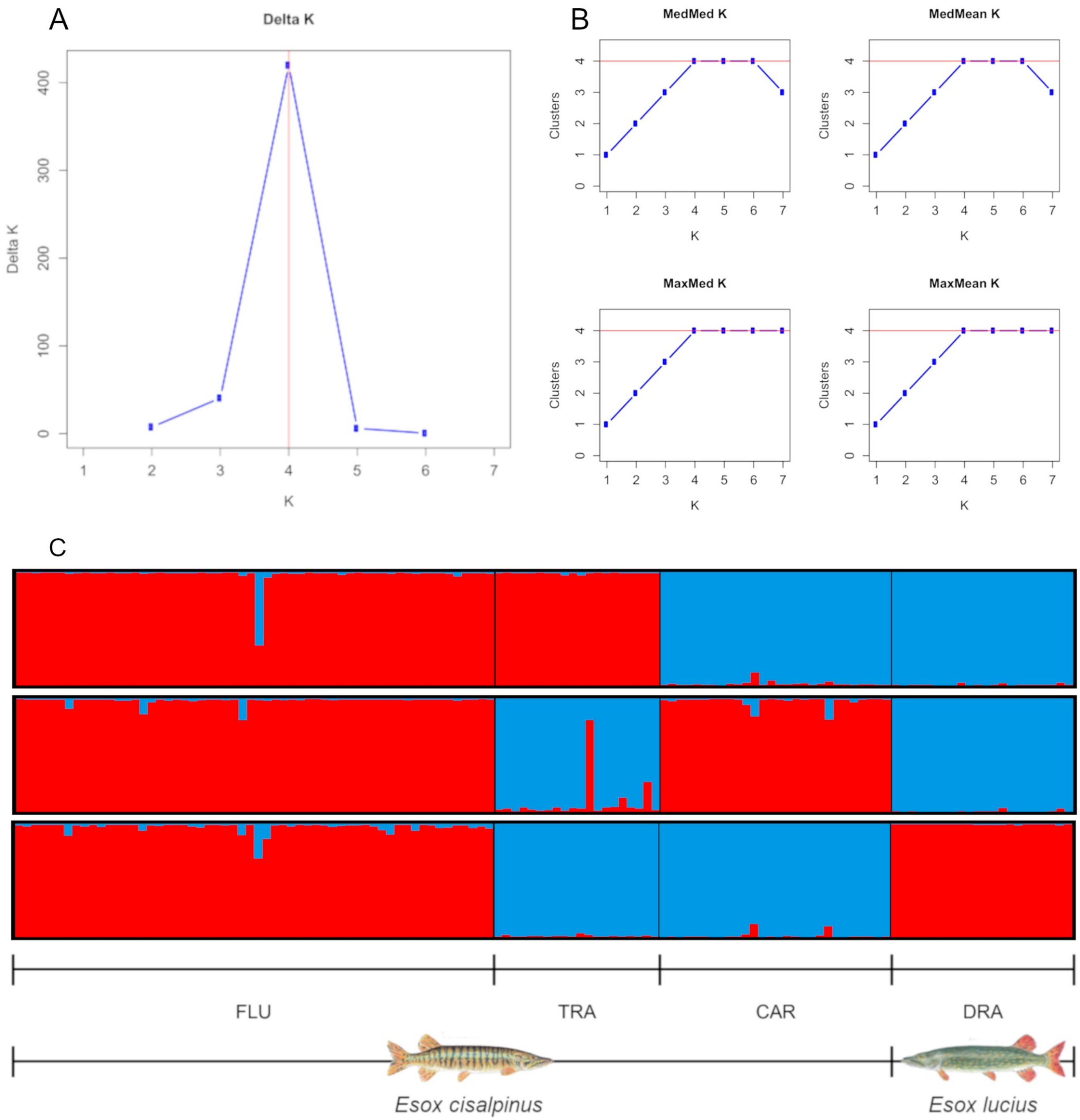


**Figure S3**. Bayesian clustering outcomes. The most likely number of clusters selected by A) the Δ*K* and B) the Puechmaille's methods is marked by the red lines in the plots. C) barplots of the clustering solutions observed at $K = 2$

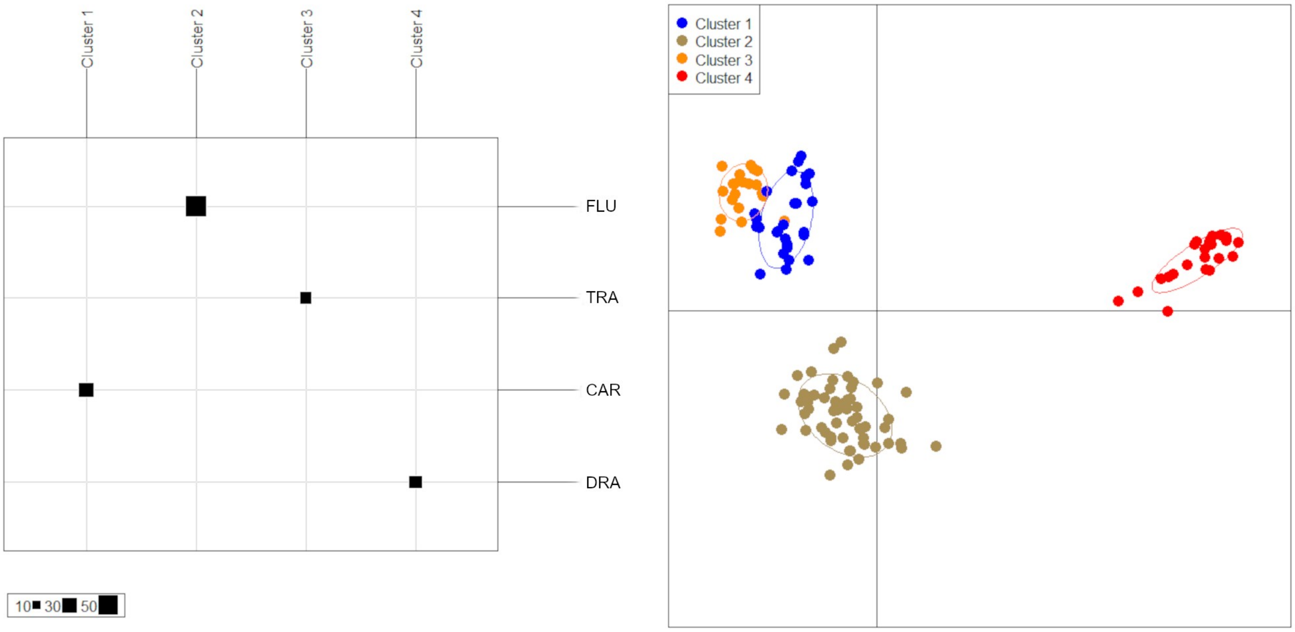


**Figure S4.** Discriminant Analysis of Principal Components (DAPC) based on K-means clustering and automated group detection. The left plot shows the perfect match between the four clusters detected using an automatic criterion (columns) and the sampling locations (rows). Square size is proportional to the number of individuals assigned to a cluster. The right plot shows the genetic structure resulting from the first (x-axis) and the second (y-axis) discrimant functions. Each dot represent an individual fish and is coloured according to the genetic cluster as well as the 95% inertia ellipses.

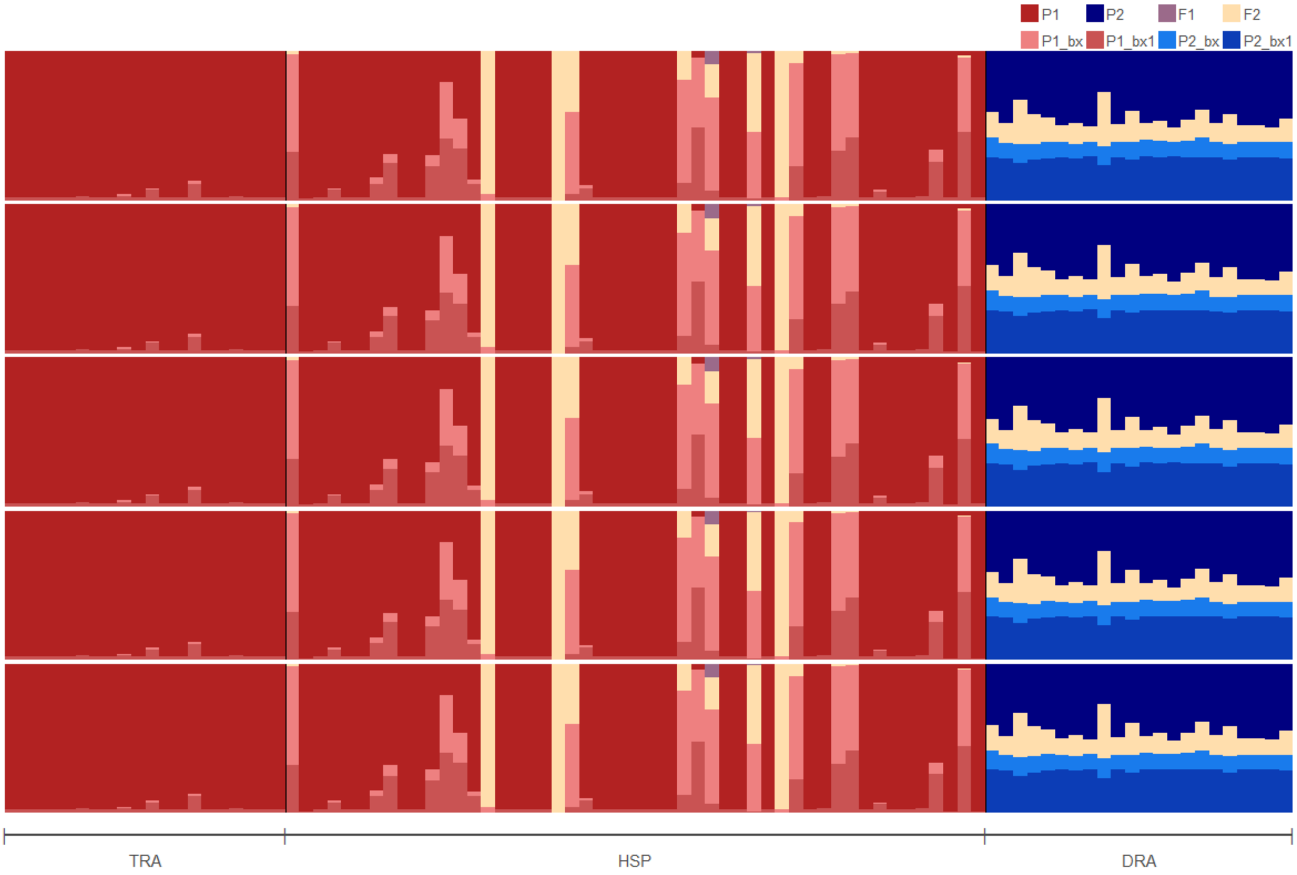


**Figure S5.** Bayesian model-based assignment of simulated individuals to different genotypic classes. Pure and hybrid Individuals in a mixed population (HSP) are simulated using real microsatellite data (Scenario 1). Genotypes from Trasimeno Lake (TRA) and Drava river (DRA) are the parental populations representing *Esox cisalpinus* and *E. lucius*, respectively. Each barplot represents the output of an indipendent run of the algorithm, in which individuals are represented by vertical bars subdivided into coloured segments. The height of each segment correspond to the assignment probability of an individual to a given genotypic class. P1 and P2 represent pure parental classes, respectively. Other classes correspond to F1 and F2 hybrids, first and second generation backcrosses between F1 with southerm (P1_bx and P1_bx1, respectively) and northern pikes (P2_bx and P2_bx1, respectively)

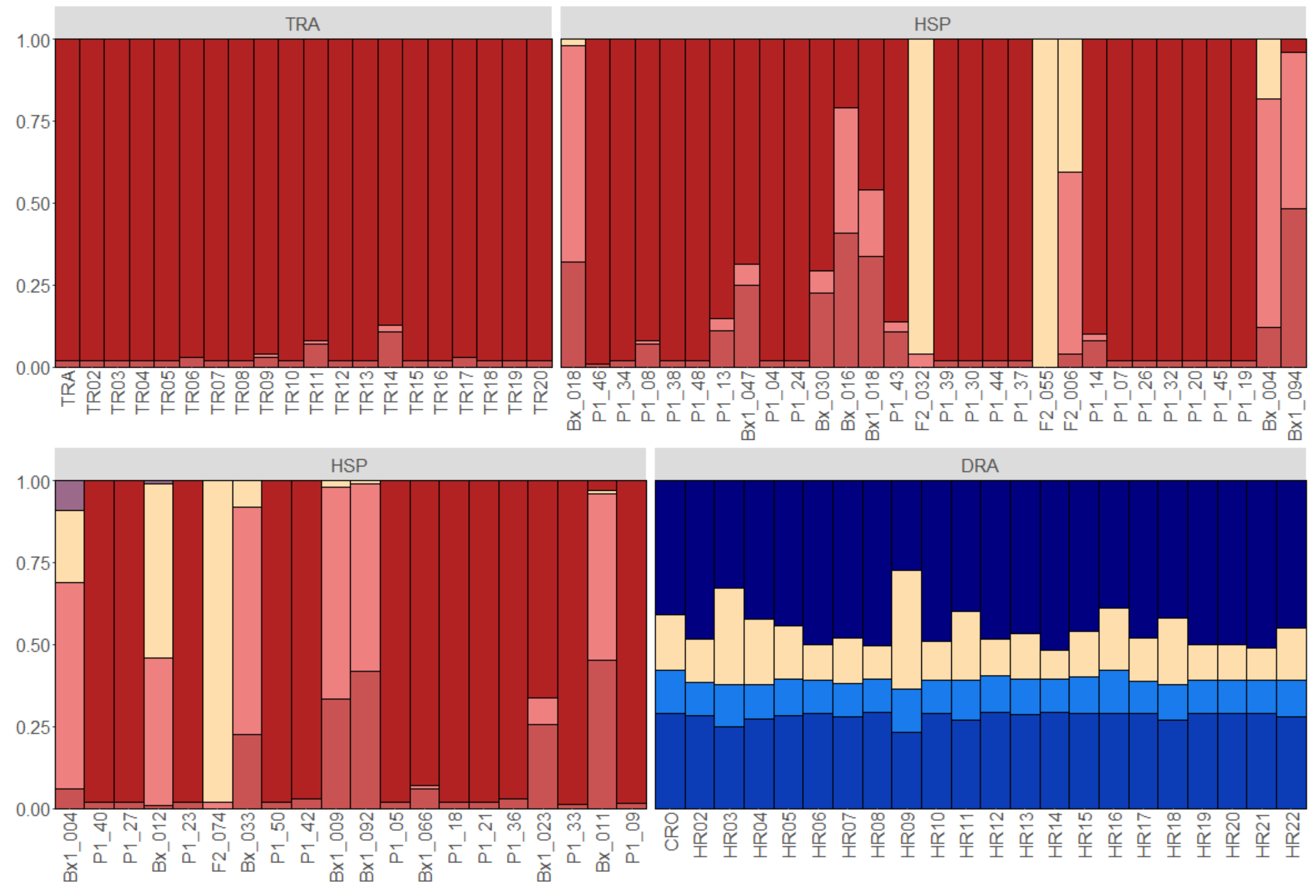


**Figure S6.** Multiline barplot obtained after merging the five independent runs illustrated in Figure S5. Each simulated individual is represented as a stacked bar and is labelled according to its true genotypic class. Height of each coloured segment is proportional to the probability of being assigned to a genotypic class. See Figure S5 for the meaning of individual prefixes in HSP and the color legend

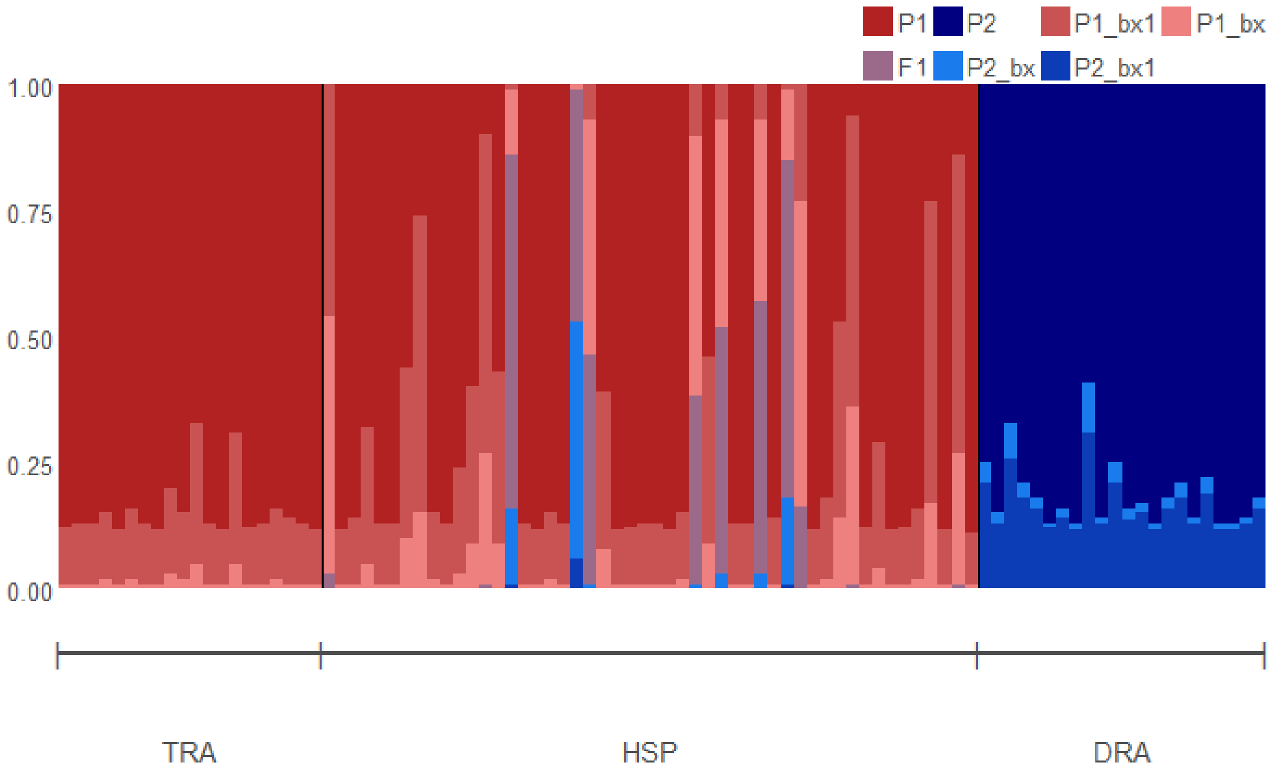


**Figure S7.** Assignment of simulated individuals to different genotypic classes based on the Expectation-Maximization algorithm. Hybrid and pure simulated individuals correspond to Scenario 1. Genotypic classes are abbreviated and coloured as in Figure S5. Note the absence of F2 genotypic class

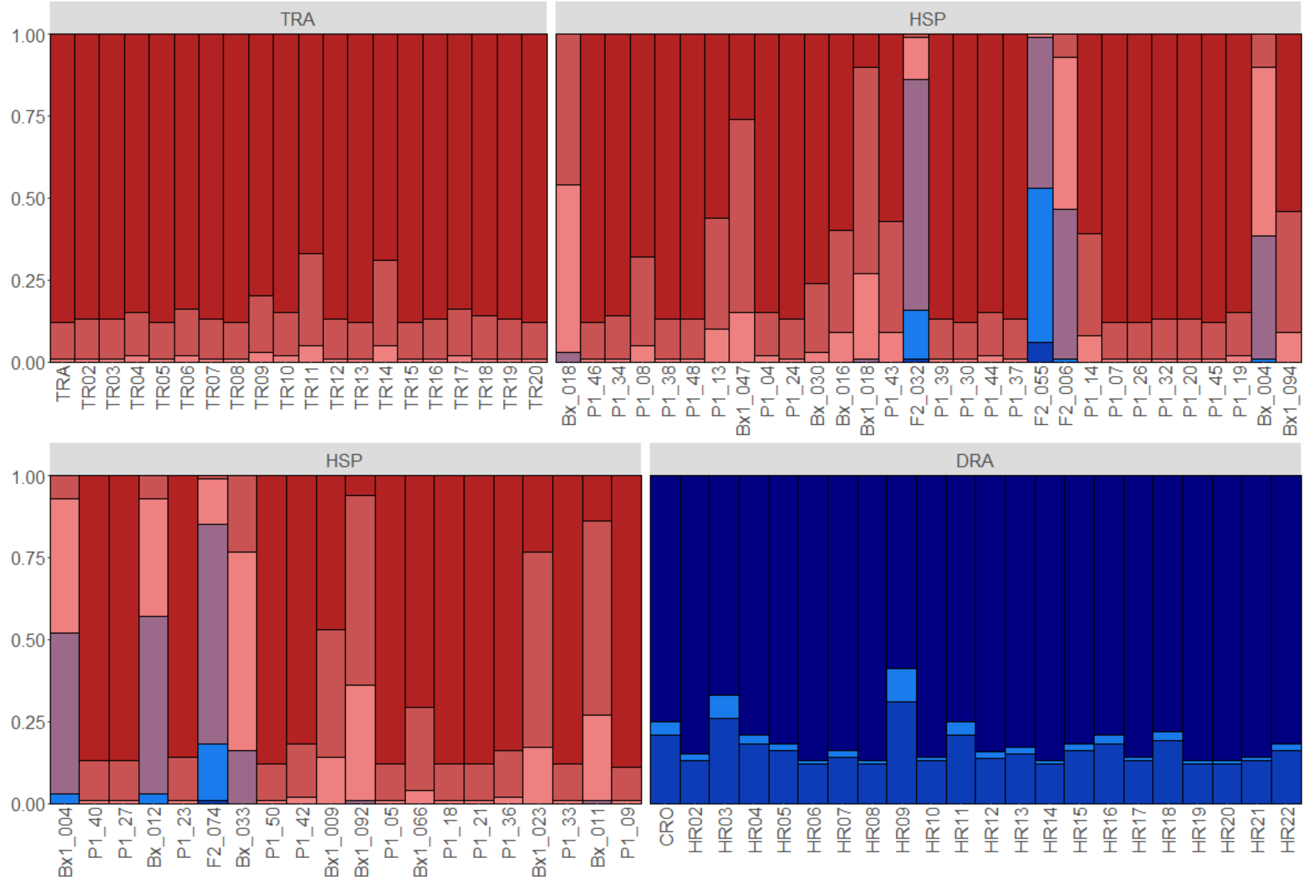


**Figure S8.** Multiline barplot of indivdual assignments illustrated in Figure S7. Although the Expectation-Maximisation algorithm cannot assign F2 hybrids to a specificic genotypic class, nontheless their genotypes are never misclassified as parental ones. See Figure S5 for the meaning of individual prefixes in HSP and the color legend

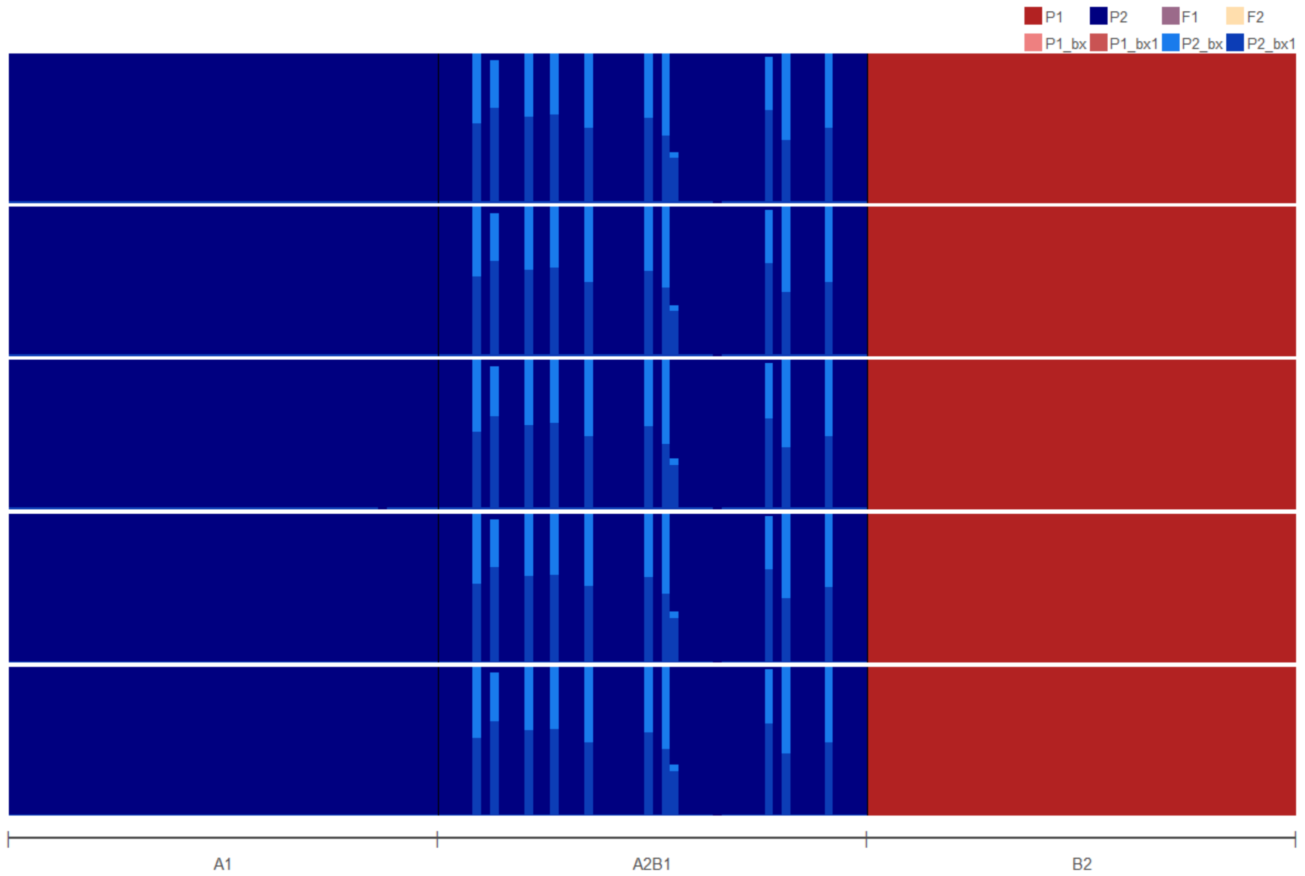


**Figure S9.** Bayesian model-based assignment of simulated individuals to different genotypic classes. The barplots show the results of five independent runs of Scenario 2, in which the evolution of 10 microsatellite loci was simulated at 2 demes each of two (sub-)populations (A1, A2, B1 and B2) under a hierarchical finite island model. Populations A2 and B1 are used to simulate introgressive hybridization into the former, but not included in the final dataset: population A2B1 is one possible outcome of this process and it is a mixture of pure individuals and backcrosses between parental (A2) and F1 hybrids. Genotypes from Pure A1 and B2 (sub-)populations are used as reference individuals in the assignment.  Genotypic classes are abbreviated and coloured as in Figure S5

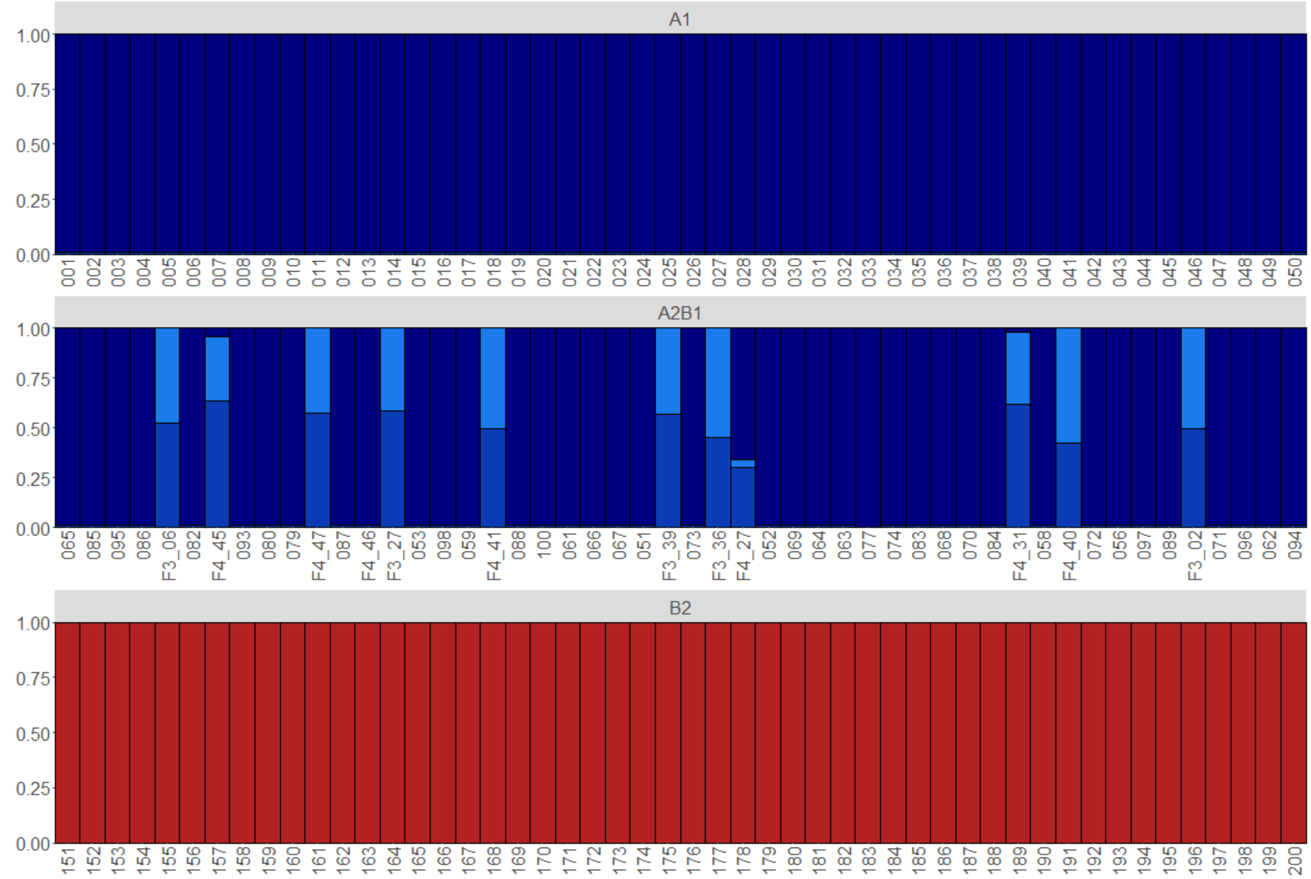


**Figure S10.** Multiline barplot obtained after merging the five independent runs illustrated in Figure S9. Hybrid individuals are marked with F3 and F4 prefixes, which correspond to first (P2_Bx) and second (P2_bx1) generation backcrosses, respectively

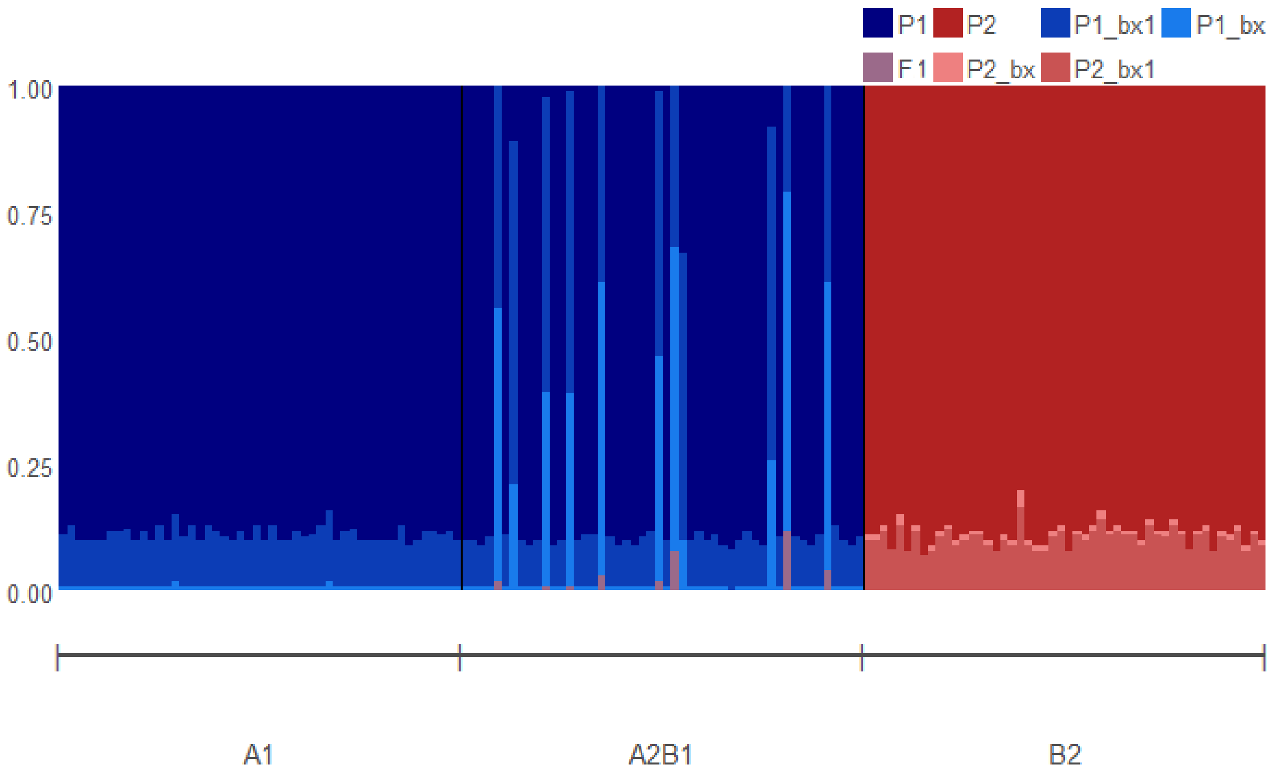


**Figure S11.** Assignment of simulated individuals to different genotypic classes based on the Expectation-Maximization algorithm. Hybrid and pure simulated individuals correspond to Scenario 2. Genotypic classes are abbreviated and coloured as in Figure S5

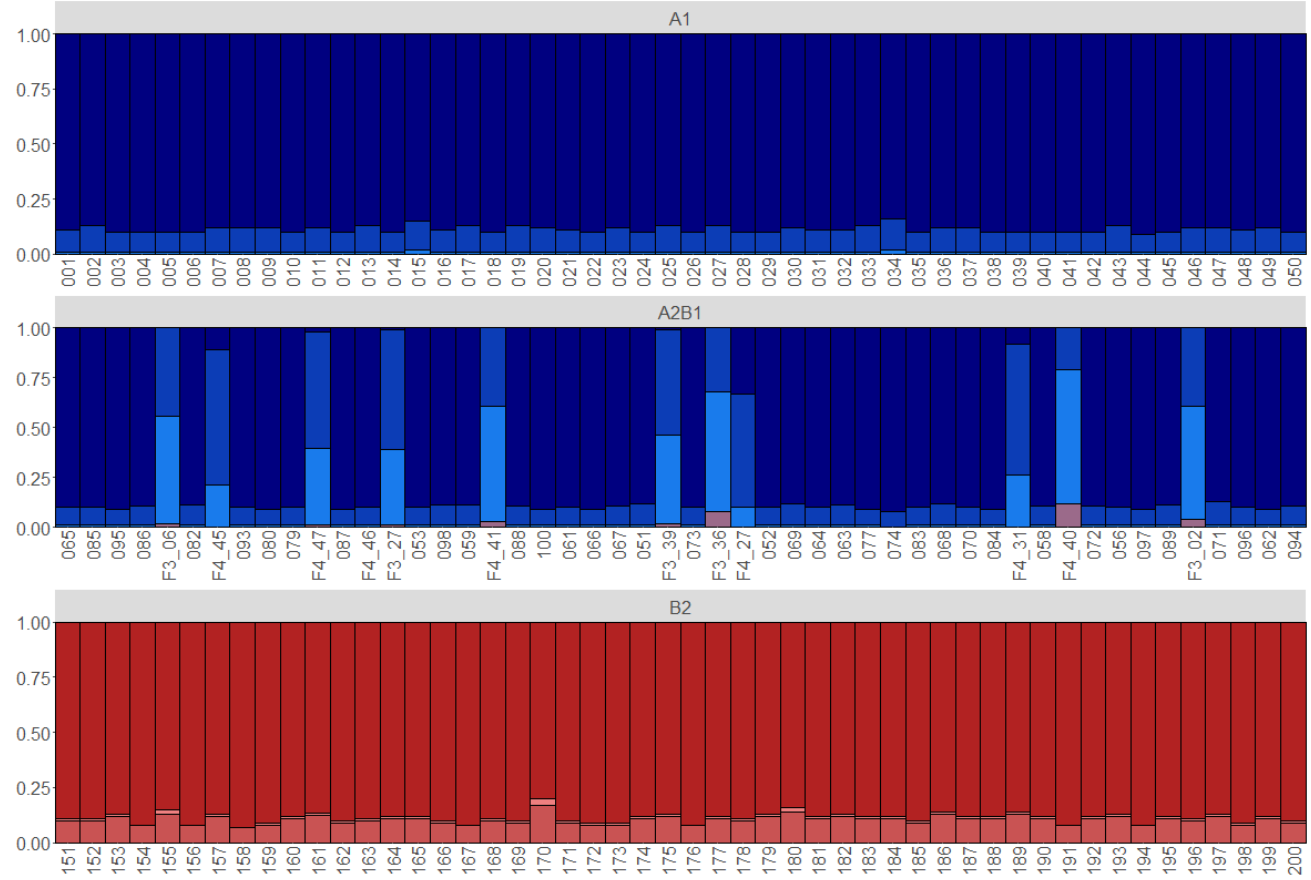


**Figure S12.** Multiline barplot of indivdual assignments illustrated in Figure S11. See Figure S5 and S10 for the color legend and meaning of individual prefixes, respectively
