# Supplementary material for: Appraising the Genetic Makeup of an Allochthonous Southern Pike Population: An Opportunity to Predict the Evolution of Introgressive Hybridization in Isolated Populations?
Source: Animals (Basel). 2023 Jan 22;13(3):380. doi: 10.3390/ani13030380 (PMC9913590; doi:10.3390/ani13030380)
Supplement: Supplementary file 1 [file animals-13-00380-s001.zip › Tables_S1-S4_SuppInfo.pdf]

Appraising the Genetic Makeup of a Southern Pike Allochthonous Population: An Opportunity to Predict the Evolution of Introgressive Hybridization in Isolated Populations?

Marco Casu[1], Ilenia Azzena[1,2], Fabio Scarpa[2], Chiara Locci[1,2], Alessio Niffoi[1], Giovanni B. Delmastro[3], Paolo Lo Conte[4], Antonio Varcasia[1], Stefano Bovero[5], Alessandro Candiotto[6], Daria Sanna[2], Piero Cossu[1]

[1] Department of Veterinary Medicine, University of Sassari, Sassari, Italy

[2]Department of Biomedical Sciences, University of Sassari, Sassari, Italy

[3]Laboratorio di Ittiologia, Museo Civico di Storia Naturale, Carmagnola, Italy

[4]Funzione Specializzata Tutela Fauna e Flora, Città Metropolitana di Torino, Torino, Italy

[5]Independent researcher "Zirichiltaggi" Sardinia Wildlife Conservation NGO, Sassari, Italia

[6]NaturaStaff Hydrobiologist, Mongardino, Italy

Corresponding author:

Piero Cossu

Department of Veterinary Medicine, Via Vienna 2, 07100, Sassari, Italy

Phone: +39 079 2280924

Email: picossu@uniss.it

Table S1. Microsatellite loci used in this study

Locus	Repeat motif	Number of alleles	Allele size range	GenBank accession number
EmaB120	(AC)14	5	227 - 235	EU108731.1
B457	(TCTA)9	17	159 - 335	AF520603.1
Elu19	(AC)23AG(AC)3	20	122 - 172	BV012656.1
Elu78	(CA)13	9	125 - 143	BV012653.1
B259	(CTAT)8	7	276 - 304	AF520598.1
EluB38	(TG)11	6	169 - 179	AY185339.1
Eluc027	(CA)10	9	140 - 164	GQ358212.1
Elu76	(AC)17	15	145 - 193	BV012657.1
Elu87	(AC)20	9	132 - 148	BV012654.1
Eluc040	(CA)16	8	227 - 249	GQ358218.1
Eluc046	(CA)23	10	180 - 208	GQ358222.1

Table S2. Summary statistics of within population genetic variation for each locus and population of *Esox cisalpinus and Esox lucius*.

	EmaB120	B457	Elu19	Elu78	B259	EluB38	Eluc027	Elu76	Elu87	Eluc040	Eluc046
FLU											
A	4	6	6	6	2	4	6	4	5	2	7
A_R	4	4.92	4.67	5.29	1.99	3.16	4.99	3.3	3.82	2	4.87
H_O	0.71	0.55	0.47	0.83	0.24	0.38	0.71	0.62	0.84	0.52	0.79
H_E	0.74	0.68	0.53	0.76	0.21	0.33	0.72	0.59	0.68	0.49	0.66
F_{IS}	0.0405	0.1923	0.1168	-0.0877	-0.1373	-0.1362	0.0174	-0.058	-0.249	-0.0545	-0.2021
F_{IS}_L	-0.1065	0.0306	-0.072	-0.2013	-0.2196	-0.246	-0.1352	-0.2692	-0.3753	-0.3096	-0.3077
F_{IS}_H	0.1978	0.3568	0.316	0.0383	-0.0727	-0.009	0.1829	0.1516	-0.1101	0.2037	-0.0915
TRA											
A	3	8	7	4	2	2	2	1	3	2	2
A_R	2.66	5.97	6.48	3.51	2	1.63	2	1	2.99	2	1.62
H_O	0.6	0.4	0.8	0.55	0.6	0.05	0.9	0	0.5	0.7	0.05
H_E	0.52	0.39	0.78	0.52	0.48	0.05	0.49	0	0.46	0.49	0.05
F_{IS}	-0.1622	-0.0224	-0.0273	-0.0654	-0.25	-0.0256	-0.8182	#N/D	-0.087	-0.4141	-0.0256
F_{IS}_L	-0.5289	-0.1879	-0.2123	-0.3674	-0.5949	-0.0957	-0.995	#N/D	-0.3546	-0.779	-0.0956
F_{IS}_H	0.2478	0.1958	0.1752	0.2709	0.1879	-0.0103	-0.595	#N/D	0.2841	0.0109	-0.0102
CAR											
A	2	5	9	3	2	4	4	2	4	4	7
A_R	1.95	4.4	6.68	2.9	2	3.27	3.3	1.52	3.38	3.98	6.12
H_O	0.14	0.57	0.5	0.54	0.29	0.57	0.54	0.04	0.21	0.68	0.71
H_E	0.13	0.46	0.52	0.55	0.29	0.55	0.42	0.04	0.31	0.67	0.65
F_{IS}	-0.0769	-0.2393	0.0416	0.0244	0.0261	-0.0407	-0.2864	-0.0182	0.3129	-0.0143	-0.1024
F_{IS}_L	-0.1639	-0.3508	-0.1223	-0.2971	-0.2488	-0.3632	-0.4414	-0.0665	-0.0994	-0.2206	-0.2771
F_{IS}_H	-0.0154	-0.1439	0.2068	0.3521	0.4525	0.2795	-0.1614	-0.0077	0.7442	0.2078	0.0977
DRA											
A	4	2	13	2	5	3	6	11	6	6	3
A_R	3.84	1.87	11.1	1.6	4.19	2.61	5.38	9.37	5.8	5.23	2.46
H_O	0.55	0.09	1	0.05	0.41	0.23	0.82	0.73	0.68	0.41	0.14
H_E	0.55	0.09	0.89	0.04	0.44	0.21	0.63	0.85	0.8	0.66	0.13
F_{IS}	0.0094	-0.0476	-0.1269	-0.0233	0.0769	-0.1055	-0.3069	0.1425	0.1473	0.3783	-0.056
F_{IS}_L	-0.2815	-0.1204	-0.1832	-0.0597	-0.1819	-0.2159	-0.4409	-0.0459	-0.098	0.0966	-0.127
F_{IS}_H	0.3398	-0.0155	-0.0902	-0.0098	0.4158	-0.029	-0.1605	0.3501	0.3891	0.6607	-0.0149

Abbreviations: A = Number of alleles; A_R = Allelic richness; H_O = Observed heterozygosity; H_E = Expected heterozygosity; F_{IS} = Inbreeding coefficient; F_{IS} L = 95% confidence interval lower limit of inbreeding coefficient; F_{IS} H = 95% confidence interval upper limit of inbreeding coefficient. FLU = Alto Flumnedosa Lake; TRA = Trasimenp Lake; CAR = Carignano-La loggia; DRA = Drava River

Table S3. Pairwise population differentiation estimated using Jost's D_{EST}. Observed values are reported below the diagonal. Lower and upper limits of 95% confidence intervals based on 10'000 bootstraps are reported within brackets above the diagonal. Populations are abbreviated as in Table S2

	FLU	TRA	CAR	DRA
FLU	—	(0.141, 0.230)	(0.203, 0.291)	(0.412, 0.505)
TRA	0.184	—	(0.196, 0.287)	(0.387, 0.464)
CAR	0.245	0.239	—	(0.532, 0.650)
DRA	0.457	0.424	0.586	—

Table S4. Range size distribution of 70 southern pikes caught in Alto Flumendosa Lake during the period 2014-2020

Class size (cm)	Frequency
0 - 10	0
11 - 20	0
21 - 30	3
31 - 40	2
41 - 50	11
51 - 60	12
61 - 70	9
71 - 80	7
81 - 90	10
91 - 100	6
101 - 110	5
111 - 120	4
> 120	1
